# Developments in nanotechnology approaches for the treatment of solid tumors

**DOI:** 10.1186/s40164-025-00656-1

**Published:** 2025-05-19

**Authors:** Jacopo Venturini, Abhijit Chakraborty, Mehmet A. Baysal, Apostolia M. Tsimberidou

**Affiliations:** 1 Department of Investigational Cancer Therapeutics, The University of Texas MD Anderson Cancer Center, Unit 455, 1515 Holcombe Boulevard, Houston, TX 77030 USA; 2 Current Affiliation: Department of Medical Oncology, Careggi University Hospital, Florence, Italy

**Keywords:** Nanotechnology, Cancer therapy, Nanoparticle-based drug delivery, Multidrug resistance, Tumor microenvironment, Targeted drug delivery, Personalized medicine, Stimuli-responsive nanoparticles

## Abstract

Nanotechnology has revolutionized cancer therapy by introducing advanced drug delivery systems that enhance therapeutic efficacy while reducing adverse effects. By leveraging various nanoparticle platforms—including liposomes, polymeric nanoparticles, and inorganic nanoparticles—researchers have improved drug solubility, stability, and bioavailability. Additionally, new nanodevices are being engineered to respond to specific physiological conditions like temperature and pH variations, enabling controlled drug release and optimizing therapeutic outcomes. Beyond drug delivery, nanotechnology plays a crucial role in the theranostic field due to the functionalization of specific materials that combine tumor detection and targeted treatment features. This review analyzes the clinical impact of nanotechnology, spanning from early-phase trials to pivotal phase 3 studies that have obtained regulatory approval, while also offering a critical perspective on the preclinical domain and its translational potential for future human applications. Despite significant progress, greater attention must be placed on key challenges, such as biocompatibility barriers and the lack of regulatory standardization, to ensure the successful translation of nanomedicine into routine clinical practice.

## Introduction

Cancer is the second leading cause of death globally, resulting in approximately 9.7 million deaths each year [1, 2]. Despite recent advancements in treatments such as surgery, radiotherapy, chemotherapy, targeted therapy, and immunotherapy, many cancers remain incurable owing to patient and tumor-related resistance mechanisms [3, 4]. Nanomedicine is a revolutionary field that combines nanotechnology with cancer therapy to improve clinical outcomes while minimizing adverse events [5–7]. Nanoparticle-based systems have been designed to improve the pharmacokinetic profile [8–10] and the actionability of anticancer drugs, optimizing their delivery [11] and overcoming the mechanisms of drug resistance [12]. Furthermore, highly sensitive and specific biosensors have been developed for cancer diagnostics [13, 14], with multifunctional nanoparticles engineered to function as both imaging and therapeutic agents, thus paving the way for theragnostic approaches [11].

Here we present a novel review of the up-to-date clinical applications of nanotechnology and the potential of their implementation through the translation of preclinical discoveries into clinical investigations. This review also explores the multifaceted role of nanotechnology in diagnostics and cancer treatment, while outlining the major challenges hindering clinical implementation.

## Methods

A narrative review of the literature was conducted using PubMed, Scopus, and ClinicalTrials.gov and employing the following keywords:"nanomedicine," "nanotechnology," "nanoparticles," "cancer therapy," "cancer diagnosis," “nanoparticle clinical trials,” “lipid-based nanoparticles,” “polymeric nanoparticle,” “biological nanoparticles,” “inorganic nanoparticles,” “advances in cancer nanotechnology.” The analysis of preclinical and clinical studies, along with high-quality reviews and meta-analyses, was guided by the coauthors’ expertise in cancer nanotechnology and personalized medicine, shaping the scope, depth, and scientific rigor of the research.

The selection of drug delivery systems remained consistent with the literature, focusing on nanoscale carriers functionalized with advanced targeting molecules for precise payload delivery. Antibody–drug conjugates were excluded. Several successful poly(lactic-co-glycolic acid) (PLGA)-based formulations with widespread clinical use were also excluded because they do not strictly fall within the nanoscale range (e.g., the leuprolide acetate depot Lupron, the Zoladex goserelin acetate depot, and Sandostatin LAR depot). For the analysis of investigational nanotechnology-based therapies in oncology, we excluded prematurely terminated studies without results, as well as trials investigating outdated regimens or already approved drugs in standard-of-care settings, ensuring a focused and representative selection of clinically relevant data.

## History of nanomedicine: from concept to cancer practice

The term nanotechnology (from the Ancient Greek νάνος, or nanos, meaning"dwarf") was first coined in 1959 by Richard Feynman during a speech envisioning the manipulation of atoms [15]. Nanotechnology refers to the development of products at the nanoscale, specifically ranging from 1 to 100 nm (nm). Nanotechnology applications are utilized in various fields, including chemistry, engineering, physics, and medicine [16, 17]. The concept of"nanomedicine"was subsequently introduced by researchers [18] to describe purposely designed systems for clinical applications that incorporate at least one component of nanometric dimensions, such as nanoparticles.

Health nanotechnology has permeated all branches of medicine, with a primary focus on cancer care, including clinical studies [19]. Over the past three decades, cancer nanomedicine research has experienced exponential growth, with several nanodevices obtaining regulatory approval worldwide and many others currently under investigation in over 200 clinical trials [20]. In 1995, the FDA approved liposomal doxorubicin (Doxil) [21], an anthracycline with improved drug targeting and reduced toxicities. During the decade from 2000 to 2010, the approval of additional polymeric, liposomal, and inorganic particles followed, with nab-paclitaxel (Abraxane) [22] being the most prominent example. Small interfering RNA (siRNA)-based nanoparticles followed [23], while immune-evading nanocarriers were developed starting in 2011 [24]. In 2017, CPX-351 (Vyxeos) [25] became the first nanomedicine to contain two drugs simultaneously. Finally, lipid nanoparticle mRNA cancer vaccines entered clinical trials in 2019 [26]. This approach gained significant attention following the widespread use of lipid nanoparticles in mRNA COVID-19 vaccines [27], and encouraging results are now being reported in patients with melanoma [28].

## Properties of nanoparticles and mechanisms of action

**Fig. 1:**
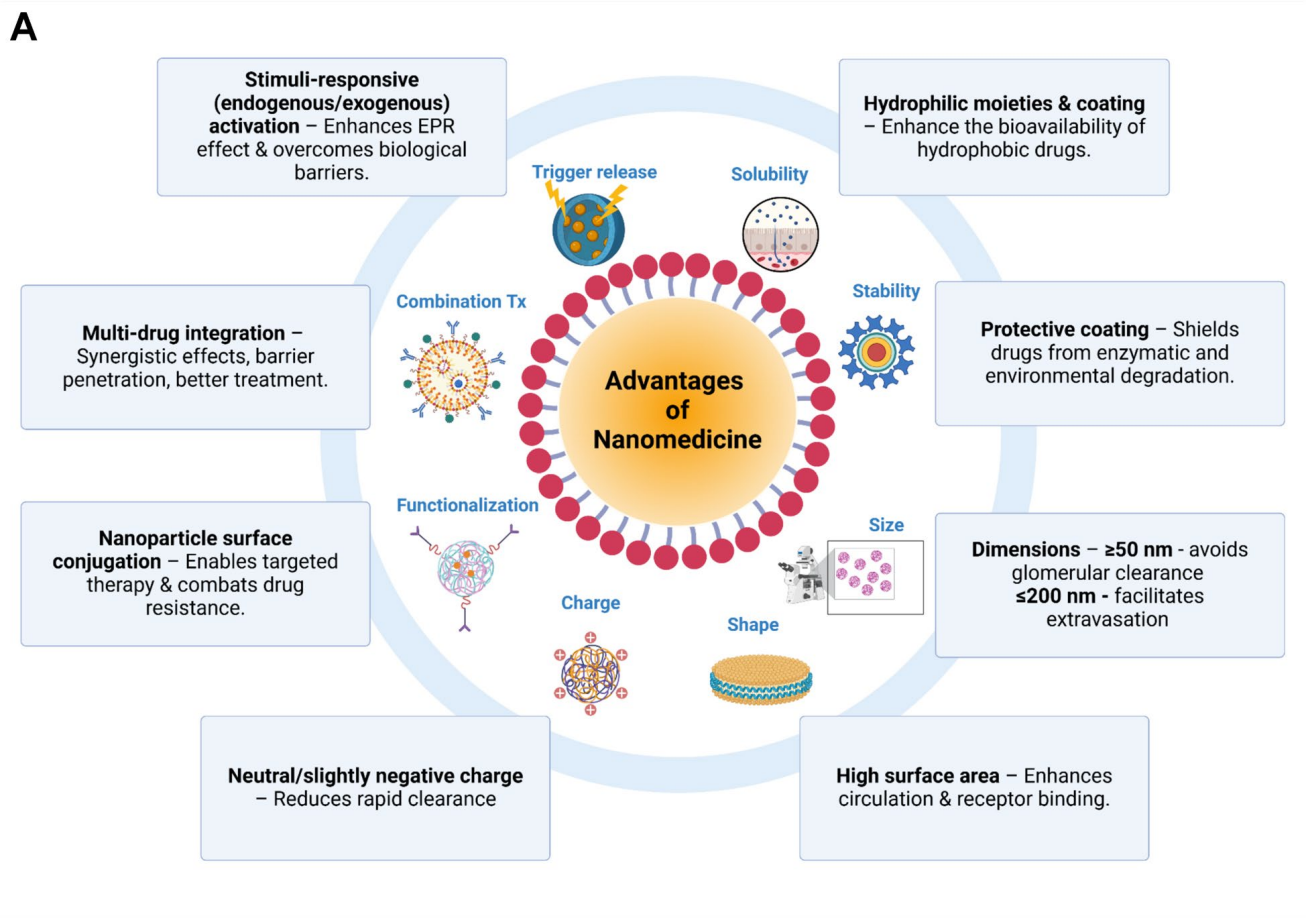

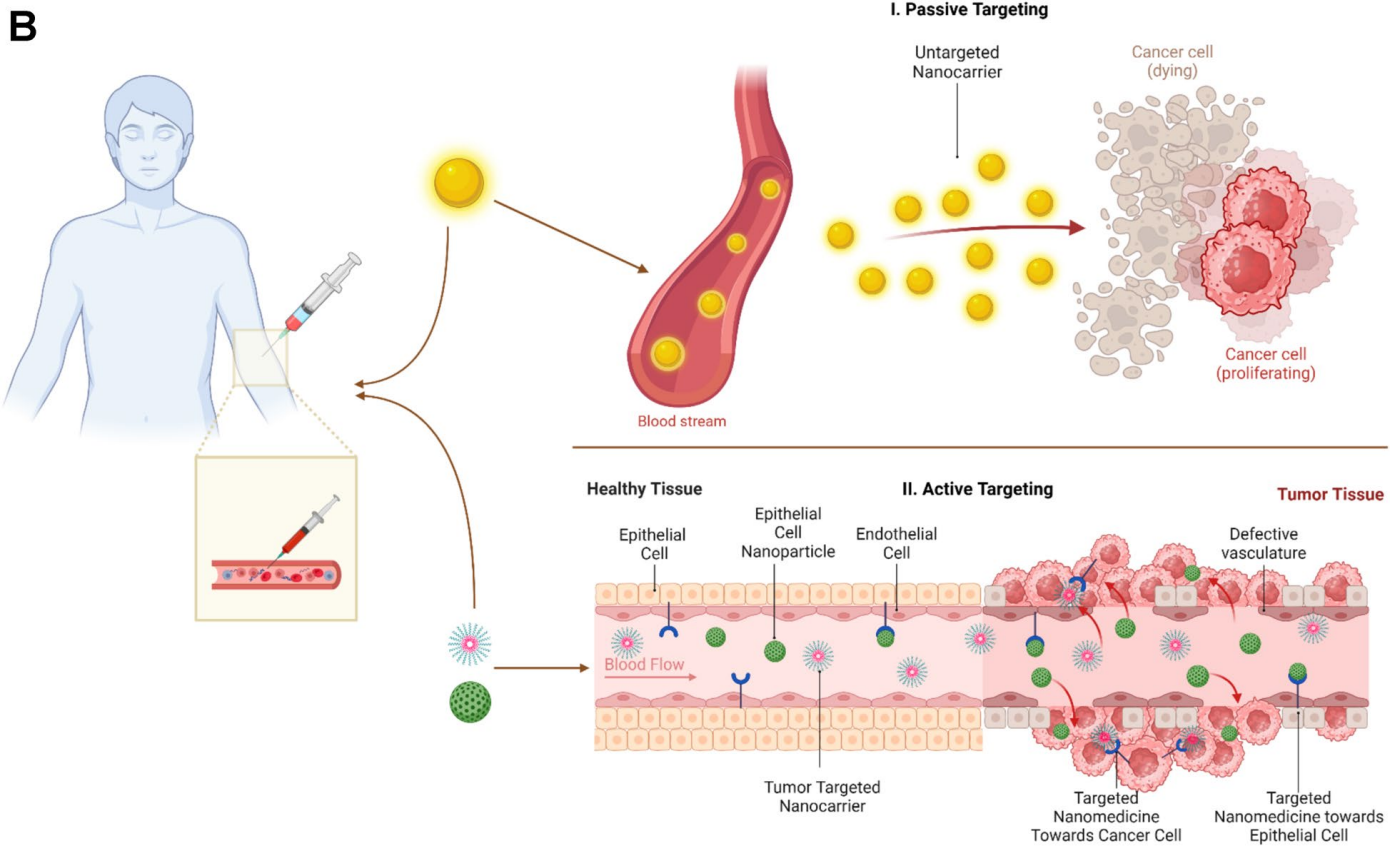
**A** Key Advantages of Nanomedicine. Key physiochemical features of nanomedicines. Nanoparticles improve bioavailability, circulation time, and targeted delivery by fine-tuning solubility, stability, size, shape, charge, and surface functionalization. Combination therapies and triggered release mechanisms enhance treatment precision, overcoming biological barriers and addressing drug resistance. EPR: Enhanced permeability and retention; nm: nanometer; Tx: Therapies. “Created with Biorender.com”. **B** Active and Passive Targeting with Nanoparticle Delivery Systems. I. Passive targeting relies on the enhanced permeability and retention (EPR) effect, where nanoparticles accumulate in tumor tissue, owing to leaky vasculature and impaired lymphatic drainage, allowing for preferential drug accumulation in solid tumors without the need for specific targeting ligands. II. Active targeting involves functionalizing nanocarriers with ligands that recognize and bind to specific receptors overexpressed on tumor cells or in the tumor microenvironment, enhancing selectivity and cellular uptake. “Created with Biorender.com”

Nanoparticles (NPs) are composed of three key components: the therapeutic payload, the core material, and biological surface modifiers [29]. These structures offer significant advantages over conventional drug delivery systems, primarily by enhancing their pharmacokinetic and pharmacodynamic profiles (Fig. 1A). From a pharmacokinetic perspective, nanoparticles are designed to improve the solubility, stability, circulation time, and delivery of the therapeutic agent payload. Thus, nanoparticle carriers afford highly hydrophobic drugs such as taxanes and anthracyclines increased bioavailability, along with protection from enzymatic degradation and environmental factors including temperature and pH fluctuations [30].

In terms of delivery mechanisms, first-generation nanocarriers—such as liposomes and polymers—began as passive targeting systems, leveraging the enhanced permeability and retention (EPR) effect [31] (Fig. 1B). The rapid and abnormal growth of tumors creates irregular and leaky blood vessels that allow nanoparticles to passively diffuse through endothelial gaps. And limited lymphatic drainage impairs the clearance of these nanoparticles, thus promoting their prolonged retention within the tumor microenvironment [32, 33]. Various factors, including size, shape, and surface characteristics, contribute to improving the efficacy of passive targeting. From a dimensional standpoint, nanodevices should ideally be kept within the range of 50 to 200 nm, thus both exceeding the 40 kDa threshold (corresponding to ~ 5 nm) for renal clearance and remaining small enough to allow for extravasation [34].

The shape of NPs is a critical factor in minimizing phagocytosis by macrophages in the liver and spleen, which constitute the reticuloendothelial system (RES). Nanodevices with very high surface area-to-volume ratios, such as rod-, discoidal-, or worm-like morphologies, have demonstrated the most advantageous and long-lasting circulation times, as they increase circulatory tumbling and receptor binding [30, 35]. Furthermore, surface characteristics also play a crucial role in the EPR effect. The optimal nanoparticle surface charge is neutral or slightly negative, as excessive positivity leads to early uptake by the negatively charged vascular endothelium and highly negative charges encourage rapid clearance by phagocytes [31].

Several limitations affect the efficacy of passive strategies, including intra- and inter-tumoral heterogeneity in the EPR and the presence of interstitial barriers. Tumor size significantly affects the vascular bed, which becomes less uniform in larger lesions, thereby confining nanoparticles primarily to the tumor’s periphery [36, 37]. Additionally, highly vascularized cancers like hepatocellular carcinoma and renal cell carcinoma display a more favorable EPR profile than other tumor types such as pancreatic cancer, which features dense stromal tissue [38]. Notably, tumor microenvironment (TME)-based obstacles represent another challenge. Tumor growth creates a hypoxic and acidic environment owing to the Warburg effect while simultaneously activating inflammatory signaling cascades that increase solid stress and interstitial fluid pressure [39]. To address these challenges, second- and third-generation nanoparticle-based therapeutics were developed, focusing on both tissue-specific and cellular-specific active targeting.

The mechanisms of second-generation nanoparticle-based therapeutics were generally focused on the functionalization of the nanocarriers. A widely adopted strategy is PEGylation, a process that involves coating the nanoparticle's surface with polyethylene glycol (PEG). PEG forms a hydrophilic and sterically repulsive layer that reduces protein adsorption (opsonization), a mechanism by which nanoparticles are marked for clearance by the mononuclear phagocyte system, particularly macrophages in the liver and spleen [30]. By minimizing recognition by immune cells, PEGylation helps the nanoparticle evade rapid clearance from the bloodstream. This stealth effect extends the circulation half-life of the nanoparticles, allowing more time for accumulation at target sites through both passive (i.e., EPR effect) and active targeting strategies. Additionally, PEG’s flexible and non-ionic nature helps prevent nanoparticle aggregation, enhancing colloidal stability in biological fluids [40]. Finally, PEGylation also enhances targeted delivery, as it is degraded by metalloproteases that are highly concentrated in the tumor stroma [41].

Subsequently, coating techniques were improved, combining nanoparticles with targeting ligands such as antibodies, nucleic acids, peptides, carbohydrates, and other small molecules to enable selective binding to tumor-specific antigens or receptors and promote active internalization processes such as endocytocis [42, 43].

NPs’ ability to selectively recognize and bind to tumor-associated antigens, and to be internalized by target cells, has been enhanced with the incorporation of immunoglobulins [44], which can be partially (antibody fragments) or completely engineered [45]. In the latter, monoclonal antibody (MoAb)-conjugated nanoparticles leverage commercially available molecules targeting well-known receptors such as the epidermal growth factor receptor (EGFR) [46], human epidermal growth factor receptor 2 (HER2) [47], prostate-specific membrane antigen (PSMA) [48], fibroblast growth factor receptor 3 (FGFR3) [49], and vascular endothelial growth factor receptor (VEGFR) [50].

Third-generation nanomedicine-based therapeutics have focused on developing triggered release techniques that enable precise drug delivery only in response to internal or external stimuli. Exogenous triggering factors like hyperthermia [51] and radiotherapy [52] have been shown to enhance nanoparticle extravasation and intratumoral distribution. Similarly, nanoparticles responsive to endogenous stimuli, such as pH shifts and protease degradation in the TME, have been engineered to overcome interstitial barriers, thus enhancing the drug’s delivery potential [53, 54]. Finally, third-generation nanotechnologies also include organelle-specific targeting strategies, where specific subcellular structures can be precisely targeted, bypassing further barriers like the endosomal/lysosomal degradation system [55].

## Nanocarriers

**Fig. 2:**
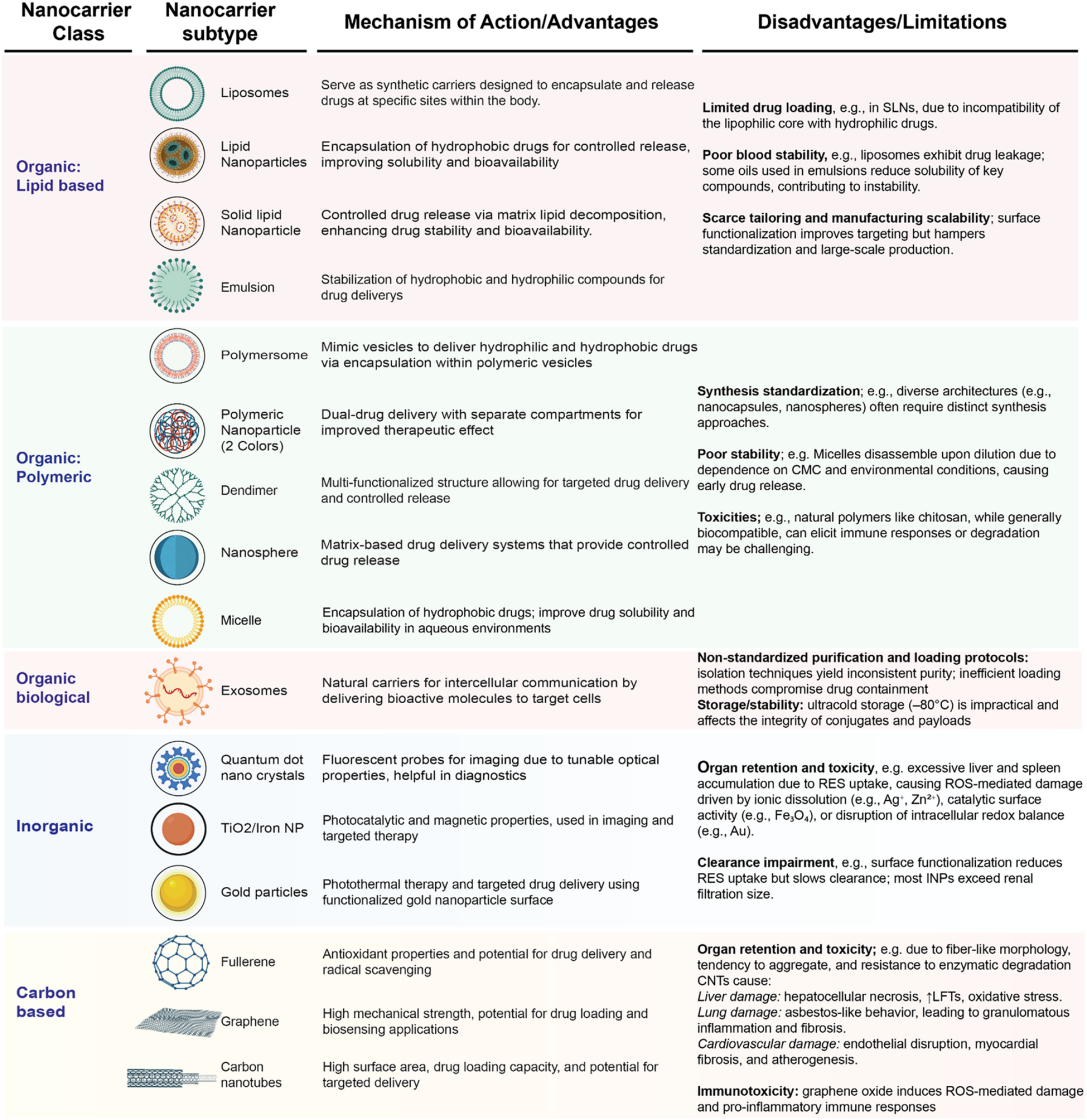
Classification of Nanocarrier Types: Mechanisms/Advantages and Disadvantages. The figure illustrates the main nanocarrier classes and subtypes and their mechanisms of action. Each class presents specific disadvantages as follows. **(a)** Among **lipid-based** systems, SLNs show limited drug loading capacity due to the incompatibility of their lipophilic core with hydrophilic drugs; liposomes suffer from poor stability and increased cargo leakage, while emulsions include oils that reduce drug solubility; surface functionalization may introduce additional manufacturing complexity and variability, limiting scalability and standardization [185, 269]. **(b) Polymeric** systems lack synthesis reproducibility due to structural diversities, which require distinct production protocols; micelles may disassemble upon dilution (i.e., low critical micelle concentration) and according to environmental conditions (pH, temperature), leading to premature drug release; natural polymers display high biocompatibility but may elicit immune responses or be resistant to degradation [270, 271]. **(c) Biological** nanocarriers lack standardized isolation protocols and the variety of existing techniques (e.g., ultracentrifugation, precipitation, chromatography, microfluidics etc.) causes inconsistent purity and therapeutic performances; drug loading methods remain inefficient: passive incubation leads to poor uptake, electroporation may damage membrane integrity; exosomal stability relies on ultracold storage (–80 °C), which is impractical for clinical use and may compromise structural and functional integrity [186, 187]. **(d) Inorganic** nanoparticles present risks of organ retention and toxicity due to their accumulation in the liver and spleen via reticuloendothelial system (RES) uptake, causing ROS-mediated damage driven by ionic dissolution (e.g., Ag⁺, Zn.^2^⁺), catalytic surface activity (e.g., Fe₃O₄), or disruption of intracellular redox balance (e.g., Au); surface modification (e.g., PEGylation) reduces RES uptake, but impairs clearance, exceeding the renal excretion threshold [272, 273]. **(e)** Among **carbon-based** nanocarriers, CNTs are associated with organ toxicity due to their fiber-like morphology, tendency to aggregate, and enzymatic resistance; these characteristics have been linked to hepatotoxicity (e.g., necrosis, oxidative stress), pulmonary inflammation and granuloma formation mimicking asbestos exposure, and cardiovascular toxicity including endothelial injury, myocardial fibrosis, and atherogenesis [174, 274–276]. CNT, carbon nanotube; NPs, nanoparticles; RES, reticuloendothelial system; ROS, reactive oxygen species; SLNs, solid lipid nanoparticles; TiO2, titanium dioxide. “Created with Biorender.com”

Nanocarriers represent a significant milestone in nanomedicine-based therapeutics. According to their distinct characteristics, they have been historically divided into the following main classes: organic (lipid-based, polymeric, and biological), inorganic, carbon-based, and other [56, 57]. Advantages and disadvantages of these nanocarriers are illustrated in Fig. 2.

### Lipid-based nanocarriers

Lipid-based nanoparticles comprise diverse subtypes such as liposomes, lipid nanoparticles (LNPs), and solid lipid nanoparticles (SLNs). Initially designed in 1964, liposomes are spherical vesicles composed of one or more phospholipid bilayers surrounding an aqueous core. Cholesterol is inserted into the lipid bilayer to decrease membrane fluidity and control the rate of drug release. This structure allows for the incorporation of hydrophilic drugs in the aqueous core and lipophilic drugs within the bilayer [58, 59]. The first liposomes had limited stability and short circulation times, which subsequently improved with surface modification techniques, thus paving the way for their widespread clinical uses [60]. PEGylation was one of the first successful functionalization processes: PEGylated doxorubicin-loaded liposomes displayed an exponential half-life increase from 3 to 55 h and had significantly reduced cardiac and bone marrow toxicity profiles compared with non-liposomal doxorubicin. However, a greater association with mucositis and palmar-plantar erythrodysesthesia was also reported, potentially due to increased drug accumulation in the eccrine glands of these skin areas [61, 62]. The effectiveness of this formulation has resulted in its approval for multiple cancer types, as detailed in the section below.

Another subset of lipid-based nanocarriers are LNPs, which are primarily used for the delivery of nucleic acids. The unique characteristics of LNPs have been pivotal in enhancing the efficacy and bioavailability of mRNA vaccines for cancer treatment, now under investigation in advanced-phase studies, with highly promising and encouraging results [63]. Indeed, unlike traditional liposomes, LNPs are composed of multiple substructures, each with a specific role: ionizable or cationic lipids to bind genetic material and facilitate endosomal escape, phospholipids and cholesterol to maintain structural integrity, and PEGylated lipids to prolong circulation time [64]. The ionizable lipids endow LNPs with a dynamic charge potential that remains near-neutral at physiological pH to minimize systemic toxicity but shifts to a positive charge in acidic environments, disrupting the endosomal membrane and permitting intracellular drug release [65].

SLNs represent a special first-generation subtype of lipid nanoparticles, characterized by the addition of solid lipids stabilized by surfactants. Due to their scarce drug loading efficiency and payload leakages, SLNs have been surpassed by the second-generation nanostructured lipid carriers, which enclose a more versatile unstructured matrix made of both solid and liquid lipids, providing more space for drug molecules [66].

### Polymeric nanocarriers

Polymeric nanoparticles are complex colloidal structures composed of natural or synthetic monomeric moieties. Depending on their structure, polymeric nanoparticles can be classified as nanocapsules, characterized by cavities surrounded by a polymeric shell enclosing a drug-containing core in solid or liquid form, or nanospheres, which consist of a matrix-like solid structure where the drug is dispersed [56]. Among natural polymers, biological proteins and polysaccharides, such as chitosan and albumin, are commonly used. Chitosan, a polysaccharide derived from crustacean shells, exhibits enhanced epithelial permeability and immunogenic properties, while albumin, owing to its human origin, provides prolonged circulation time and accumulation within the abnormal tumor vasculature [67].

Though also considered part of the biological nanocarrier class, nab-paclitaxel represents a significant breakthrough in natural polymer-based nanotechnology. It consists of a colloidal solution in which paclitaxel is non-covalently bound to albumin, thus avoiding the need for the Cremophor EL surfactant used in traditional paclitaxel formulations. Serum albumin offers increased cancer tropism not only through the EPR effect but also by leveraging endothelial transcytosis. This process initiates with caveolin-mediated drug internalization upon albumin binding to gp60 receptors on endothelial cells and culminates in drug accumulation within the TME due to its interaction with the tumor-secreted protein SPARC (secreted protein, acidic and rich in cysteine) [68, 69]. Furthermore, the absence of Cremophor EL prevents drug entrapment within the plasma compartment, enhancing paclitaxel's bioavailability. Nab-paclitaxel also significantly reduced adverse events such as myelosuppression, neurotoxicity, and hypersensitivity reactions commonly associated with the surfactant [70, 71]. Nab-paclitaxel-based therapy has been extensively investigated in many clinical trials and has demonstrated antitumor activity across various tumors [70, 71].

Various synthetic polymers have received FDA approval owing to their high biodegradability and biocompatibility, including PLGA-based formulations, which are produced through the copolymerization of distinct lactide and glycolide monomers [72]. One notable example is Eligard, which combines the luteinizing hormone-releasing hormone (LHRH) analog leuprolide acetate with a PLGA polymer matrix to create a depot system for sustained drug release, providing long-term suppression of testosterone levels in patients with advanced prostate cancer [73].

Finally, polymeric nanoparticles can be further categorized into different types of polymer combinations: polymersomes, dendrimers, and micelles. Polymersomes are vesicle-like nanostructures with bilayer membranes composed of amphiphilic block copolymers, which are biomimetic analogs of natural phospholipids, thus allowing for hydrophobic and hydrophilic drug carriage [72]. Dendrimers are highly organized, hyperbranched polymers with multiple functional groups on their surface, offering remarkable drug-loading capacity. Their multicompartmental design enables the simultaneous delivery of multiple therapeutic agents, conferring exceptional multitargeting potential. Hydrophobic drugs, for example, can be encapsulated through ionic interactions, hydrogen bonding, hydrophobic interactions, and even covalent bonding [74]. Polymer micelles are nano-colloidal structures formed by the self-assembly of amphiphilic block copolymers in aqueous solutions. Their structure includes a hydrophobic core for encapsulating drugs and a hydrophilic shell for particle stability. However, their formation and stability are closely dependent on the critical micelle concentration, which must be low to ensure resistance to dilution in physiological conditions, thereby prolonging their circulation time and effectiveness [75].

### Inorganic nanocarriers

Inorganic nanocarriers are composed of metals, metal oxides, and carbon-based nanomaterials [76]. Gold nanoparticles (AuNPs) feature low immunogenicity, reliable synthesis, high surface area ratio, and versatile surface chemistry, making them ideal for combination and targeted drug delivery. AuNPs can be synthesized in various shapes (i.e., sphere, rod, star, and cage-like morphologies) using chemical, physical, or biological methods. Chemical AuNP synthesis, such as with the Turkevich method, which reduces AuCl4 using tannic or ascorbic acid, and physical techniques (e.g., radiation, laser ablation) rely on high temperatures, pressure, and toxic reagents, whereas biological approaches (e.g., using microalgae, bacteria, fungi, or plants) offer eco-friendly and biocompatible alternatives [77, 78].

AuNPs feature substantial surface modification potential, encompassing functionalization with specific targets (e.g., eugenol and hyaluronic acid) and the surface plasmon resonance (SPR) phenomenon. This phenomenon occurs in response to incident electromagnetic radiation, forming free electrons at the surface of noble metal nanoparticles that resonate and lead to significant light absorption and scattering. This resonance often occurs at specific wavelengths that are determined by the nanoparticle's material properties and dimensions. This enables AuNPs to efficiently absorb and scatter light, particularly within the near-infrared (NIR) biological window (650–1100 nm), yielding promising results in the field of photothermal therapy (PTT) [79]. Upon excitation by NIR light, the SPR effect in AuNPs induces localized heating (resulting from the optical absorption of incident light) that increases the kinetic energy of surface electrons and leads to efficient thermal energy generation. This targeted hyperthermia can induce apoptosis in cancer cells, while minimizing damage to surrounding healthy tissue [80, 81]. The SPR properties of AuNPs have also been applied to biosensing. SPR-based biosensors show high sensitivity in capturing refractive index changes near the nanoparticle surface, which result in measurable shifts in resonance wavelength, permitting real-time, label-free detection of biological interactions [82, 83].

Mesoporous silica nanoparticles (MSNs) consist of an amorphous silicon dioxide wall structure with 2- to 50-nm pores suitable for accommodating drugs of various molecular shapes [84]. MSNs are highly biocompatible and can be efficiently synthesized through soft templating, using surfactants like cetrimonium bromide to form micelle-based templates, or hard templating, with metal oxides or polymer beads [85]. Their high surface area provides ample sites for functional group attachment through silanol bonds (also known as “gatekeepers”), which are cleavable only in response to specific environmental stimuli, naturally predisposing MSNs to pH-dependent or reactive oxygen species (ROS)-dependent drug release [86].

Iron oxide nanoparticles (FeNPs) are emerging as promising therapeutic and diagnostic nanoscale carriers due to their “superparamagnetism,” or property of becoming magnetized under an applied magnetic field. Although naturally produced by certain bacteria within organelles known as magnetosomes, these superparamagnetic iron oxide NPs (SPIONs) are commonly synthesized chemically for greater cost effectiveness and scalability [87]. SPIONs exhibit a unique ability to respond to external magnetic fields while remaining non-magnetic in their absence. This property makes them particularly advantageous in hyperthermia treatment for cancer. When exposed to an alternating magnetic field, the magnetic moments of SPIONs undergo rapid rotational motion and generate frictional forces at the molecular level, leading to the dissipation of energy in the form of heat, which in turn increases the temperature of cancerous tissues, promoting cellular apoptosis and necrosis while sparing surrounding healthy tissues [88]. Notably, alternating magnetic field-free FeNP showed anti-cancer effects by inducing ferroptosis, a non-apoptotic cell death mechanism in which intracellular iron accumulation impairs the cell's scavenging defenses by inhibiting glutathione peroxidase [89].

### Carbon-based nanocarriers

Carbon-based nanomaterials exhibit remarkable potential due to their diverse structural forms. According to the type of *sp* hybridization, NPs can be classified as two-dimensional, flat structures like graphene, and one-dimensional, hollow structures such as carbon nanotubes [14]. Graphene’s 2D structure offers a large binding surface area on its hydrophobic basal plane for efficient loading of anticancer agents through hydrophobic interactions or conjugate reactions, while hydrophilic drugs can attach at its edges via electrostatic interactions and hydrogen bonding [14]. Graphene oxide (GO), a graphene derivative, permits conjugation with functional groups (e.g., -COOH, -OH, -O-), thus increasing its actionability potential. Moreover, graphene and its derivatives exhibit broad-spectrum light absorption, from ultraviolet to NIR regions, enabling their use in light-driven therapies like photothermal and photodynamic therapies [90].

Carbon nanotubes are cylindrical hollow structures (0.4–100 nm in diameter and up to several micrometers in length) composed of rolled graphene sheets and can be single-walled (SWCNTs) or multi-walled (MWCNTs) depending on the number of concentric layers. Their needle-like structure allows for efficient penetration through cellular barriers, while their strong light absorption in the NIR region facilitates PTTs [14]. As multifunctional platforms, carbon nanotubes have also been successfully applied to cancer imaging, leveraging their ability to transform laser energy into acoustic signals (i.e., photoacoustic effect) [91].

Despite their remarkable potential, however, the application of inorganic nanocarriers in clinical practice has several pharmacokinetic limitations. Functionalization approaches to address these limitations are under investigation, as will be further discussed.

### Other nanocarriers

Among the new nanocarrier class, biological nanoparticles with enhanced biocompatibility have emerged. Extracellular vesicles, cell membrane-derived particles that were formerly considered cellular waste, have garnered interest. Among extracellular vesicles, exosomes represent the most studied subset; exosomes range from 30 to 150 nm and are secreted by both healthy and cancer cells [92, 93]. These vesicles play critical roles in intercellular communication, offering potential applications across multiple therapeutic and diagnostic domains. Indeed, exosomes hold promise for non-invasive cancer diagnosis through the detection of their biochemical components, targeted drug delivery by leveraging their complete biocompatibility, and cancer immunotherapy by modulating the complex interplay with the immune system [92, 93].

## Nanotechnology in cancer therapy

The application of nanotechnology in cancer therapy includes FDA-approved drugs (Table 1) and investigational agents (Table 2).

**Table 1:** FDA-approved nanotechnology-based drugs and selected trials

Drug	Nanocarrier Type	Tumor Type	FDA Approval Year	Selected Pivotal Studies	Comments
Doxil (liposomal doxorubicin)	PEGylated liposomal nanoparticles	Ovarian	1999 2005	F M Muggia et al. [96], 1997 Gordon AN et al. [97], 2004	First FDA-approved nanodrug, extends circulation time and minimizes cardiac adverse events. Accelerated approval, 1999; full approval, 2005
		Multiple myeloma	2007	Orlowski RZ et al. [98], 2007	Full approval, 2007
		AIDS-related Kaposi sarcoma	1995	Northfelt DW et al. [94], 1997 Stewart Set al [95], 1998	Accelerated approval, 1995
DaunoXome (daunorubicin citrate liposome injection)	Liposomal	AIDS-related Kaposi sarcoma	1996	PS Gill et al. [209]., 1996	Clinical trials show efficacy, improved safety, and enhanced targeting with reduced toxicity via liposomal formulation Full approval, 1996
Abraxane (Nab-paclitaxel)	Albumin-bound nanoparticles	Breast	2005	William J Gradishar et al. [100], 2005 Nuhad K Ibrahim et al. [101], 2005	First nanotechnology-approved paclitaxel, enhancing solubility and reducing solvent-based toxicity Full approval
		Pancreatic	2013	Von Hoff et al. [103], 2013	
		NSCLC	2012	Socinski MA, et al. [102], 2012	
Eligard (leuprolide acetate)	Polymeric mixture	Prostate	2002	Perez-Marreno R [210], 2002 Chu FM et al. [211], 2002	The polymeric mixture permits gradual, subcutaneous drug release at a controlled rate Full approval, 2002
Neulasta (pegfilgrastim)	PEGylated protein	Supportive therapy (chemotherapy-related neutropenia)	2002	F A Holmes et al. [109], 2002 M D Green et al. [110], 2003 CL Vogel et al. [111], 2005	PEGylated G-CSF used to reduce neutropenia risk in cancer patients receiving chemotherapy. Improves neutrophil recovery and reduces infection risk
DepoCyt (cytarabine liposome)	Liposomal cytarabine	Lymphomatous meningitis	1999	Glantz et al. [107], 1999 Glantz et al. [108], 1999	Prolonged drug release in cerebrospinal fluid, enhancing efficacy in meningitis cases
Onivyde (pegylated liposomal irinotecan)	Liposomal irinotecan	Pancreatic	2015 2024	Wang-Gillam et al. [105], 2016 Wainberg ZA et al. [106], 2023	Extended drug retention reduces systemic toxicity, supporting better therapeutic outcomes. Approval for the refractory setting, 2015, was prior to the official publication; as 1 st line treatment, 2024
VyxEOS (daunorubicin and cytarabine liposome)	Liposomal formulation combining daunorubicin and cytarabine	Acute myeloid leukemia	2017	Lancet et al. [25], 2018	Enhances synergy of drugs within liposomes for targeted AML therapy. Approval prior to official publication, 2017
Oncaspar (pegaspargase)	PEGylated liposome	Acute lymphoblastic leukemia	1994 2006	Ettinger LJ et al. [212], 1995 PA Dinndorf et al. [213], 2007	A PEGylated form of asparaginase designed to improve pharmacokinetics, reduce immunogenicity, and enhance therapeutic outcomes 1994 and 2006 approval dates correspond to two different disease settings. 1994’s approval was prior to official publication
Emend (aprepitant)	Liposomes, polymer-based nanoparticles	Chemotherapy-related nausea and vomiting	2003	Hesketh PJ, et al. [112], 2003	Antiemetic to prevent nausea and vomiting during chemotherapy, improving cancer treatment outcomes—nanoparticle formulations to enhance its bioavailability and targeted delivery

**Table 2:** Selected Phase 1,2,3 trials with nanomedicine-based drugs

Study ID, year	Phase	Tumor type	Setting	No. of pts	Treatment	Primary endpoint	Study Results	Comment
NCT01644890, 2019	3	Breast	1 st line	211	NK105 (micellar paclitaxelNP) vs paclitaxel	PFS (non-inferiority margin: 1.21)	mPFS:8.4 vs 8.5 mo. (adjusted HR: 1.255; CI: 0.989–1.592) mOS: 31.2 mo vs 36.2 mo (adjusted HR: 1.197; 95% CI: 0.885–1.620) ORR: 31.6% vs 39.0% TRAEs: PSN cumulative incidence NK105 vs paclitaxel (p < 0.0001)	Primary endpoint not reached
NCT0158342 (Geparsepto), 2019	3	Early breast cancer	Neoadjuvant	1229	Nabpaclitaxel $\to$ epirubicin + cyclophosphamide vs paclitaxel $\to$ epirubicin + cyclophosphamide	pCR	pCR: 38% vs 29% (non adjusted p = 0.00065) pCR TNBC: 48.2% vs 26.3 (p < 0.001) 4 y DFS rate: 84.0% *vs* 76.3% (HR 0.66; 95% CI, 0.51 to 0.86; p = 0.002), 4y OS rate: 89.7% *vs* 87.2 (HR 0.82; 95% CI, 0.59 to 1.16; p = 0.260) Grade ≥ 3 TRAEs: higher PSN (p < 0.001) and anemia (p = 0.048) for nab-paclitaxel vs paclitaxel Dose reduction rate: 30% vs 12 (p < 0.0001)	The greatest efficacy in the TNBC population -confirmed by the ETNA trial – must be weighed against higher toxicities. Nab-paclitaxel stands as an alternative in case of hypersensitivity to paclitaxel
NCT01822314 (ETNA trial). 2018	3	Early HER2 negative breast cancer	Neoadjuvant	814	Nabpaclitaxel $\to$ investigator’s choice anthracycline-base Tx vs paclitaxel $\to$ investigator’s choice anthracycline-base Tx	pCR	pCR: 22.5% vs 18.6% (OR 0.77; 95% CI, 0.52–1.13; P =.19) ≥ 1 SAE: 16% vs 11.3% AE: PSN G ≥ 3 4.5% vs 1.8% Multivariate analysis: TNBC vs luminal B-like OR for pCR 4.85 (95% CI, 3.28–7.18)	Nabpaclitaxel performed numerically than paclitaxel. The major benefit was noted in the TNBC population
NCT00617981 (HEAT study), 2018	3	HCC	Non operable BCLC-A and B	701	RFA + LTLD vs RFA	PFS	mPFS: 13.9 vs 13.9 mo (HR 0.96, 95% CI 0.79–1.18; p = 0.71) mOS: 53.4 vs 53.7 (HR 0.98, IC 95% CI, 0.80–1.20; p = 0.82) OS RFA dwell time ≥ 45 min and solitary lesion: HR: 0.63, (95% CI: 0.41–0.96, p < 0.05) All grade TRAEs: 83% vs 35%	The time correlation suggests the role of RFA-mediated heat in enhancing drug release. The benefit in solitary lesions favors an earlier-stage approach
NCT01492101 (BEACON trial), 2015	3	Breast cancer	Secondor further lines	852	NTRK102 (etirinotecan pegol/PEG irinotecan) vs physician's choice regimen	OS	mOS: 12.4 vs 10.3 mo (HR 0.87, 95% CI 0.75–1.02; p = 0.084) mOS with brain mets: 10 vs 4.8 mo; HR 0.51, p < 0.01) Grade ≥ 3 TRAEs: 48% vs 63% (p < 0.001)	Its trend in clinical benefit and the lower toxicities suggest comparable applicability to other later-line regimens
NCT02379845, 2019	2/3	Soft tissue sarcoma	Neoadjuvant	185	NBTXR3 (hafnium oxide (HfO2) nanoparticle activated by RT) vs RT	pCR rate	pCR: 16% vs 8% (p = 0.044) R0 resection:77.0% vs 64.0% (p = 0.042) ORR: 6.9% vs 10.1% (p = 0.863) 24-mo DMFS rate: 33.3% vs 26.2% SAE: 10.1% vs 5.6%	The long-term safety results and the efficacy in local and distant recurrence support the use of this combination
NCT03897881, 2024	2	Melanoma(stage IIIB–IV)	Adjuvant	157	mRNA-4157 (LNP-based) + pembrolizumab	RFS	18-mo RFS rate: 79% vs 62% (HR 0.561, 95% CI 0,309–1,017; p = 0.053). 18-month DMFS: 92% vs 77% (HR 0.347, 95% CI 0.145–0.828; p = 0.013) TRAE G3: 12% vs 0% No G4-5 reported	The DMFS benefit is promising, due to its probable OS surrogacy for melanoma. TRAEs were higher in the combination group, but of milder grade. Phase 3 trial (NCT05933577) ongoing
NCT04381910, 2024	2	Extended SCLC	1 st line	66	LY01610 (liposomal Irinotecan) 60 mg/m2, 80 mg/m2, 100 mg/m2	ORR and DCR	ORR: overall 32% (CI 95%: 21–44) mDoR: 5.2 mo. (CI 95%: 3.0–8.3) mPFS: 4.0 mo. (CI 95%: 2.9–5.5) mOS: 9.7 mo. (95% CI: 7.2–12.3)	The 80 mg/m2 dose showed better outcomes
NCT02680535, 2024	2	Localized prostate cancer	Focal therapy	46	Gold nanoshell-directed photothermal ablation	CR assessed combining MRI and MR/US fusion biopsy	3-mo CR: 66% 12-mo CR: 73% 12-mo PSA reduction vs basal: p < 0.0001 AEs: No G3-4 adverse events Sexual functionality restored at 12 months	First-in-human trial of gold nanoparticle-mediated focal ablation. Demonstrated feasibility and promising tumor control with minimal toxicity
NCT03579771 (NEOGAP), 2023	2	High-risk intrahepatic BTC	30	Neoadjuvant	Nabpaclitaxel + cisplatin + gemcitabine	Tx and surgery completion rate	Tx and surgery completion rate: 73% (90% CI 57–86; p = 0.008) DCR: 90% mRFS: 7.1 mo mOS: 24 mo Grade ≥ 3 TRAEs: 33% (most common: neutropenia, diarrhea)	OS was NE for the patient who underwent surgery This perioperative strategy is feasible and safe
NCT04831320, 2023	2	HNSCC	15	2nd line	Nab-paclitaxel + nivolumab	ORR	ORR:47%	Enrollment to the second phase is ongoing
NCT03463265, 2023	2	High-grade glioma, glioblastoma	62	1 st line	Arm A: Cohort 1: ABI-009 (nab-sirolimus) + TMZ; Cohort 2: A. + bevacizumab; Cohort C: A. + lomustine; Cohort D: A. + marizomib; Arm B: ABI 009 + temozolomide and RT	ORR	Arm A ORR: 0% for all cohorts mPFS: Cohort 1: 1.7 mo. (CI 95%, 1.3 to NA); Cohort 2: 11 mo. (CI 95%, 5.2 to NA); Cohort 3: 3.1 mo. (CI 95%,1.7 to 9.2), 3.8 mo. (CI 95%,1.4 to NA), 1.7 mo (CI 95%, 0.9 to 3.5) Arm B ORR: 11.5% (CI 95%, 2.4 to 30.2) mPFS 7.5 mo (CI 95%, 6.2 to 14.4) mOS: 13.3 mo (7.9 to 23.2) mOS: Cohort 17.2 mo (CI 95%, 2.7 to NA), 13.8 mo. (5.2 to NA) 6.8 (CI 95%, 1.7 to 13.1), 7.5 mo (CI 95%, 5.4 to NA) 6.7 mo. (CI 95%, 1.7 to 9.2)	Primary endpoint not reached. Best results in the combination arm with RT
NCT02573493, 2021	2	Locally advanced HNSCC	80	Induction therapy	Nab-paclitaxel + cisplatin $\to$ CRT vs nab paclitaxel $\to$ CRT	cCR	cCR: Arm 1: 70%; Arm 2: 20% Grade ≥ 3 TRAEs: Arm 1: 58% and Arm 2: 43%	Results suggest a possible use in case of ineligibility to standard Tx
NCT02716038, 2020	2	Stage Ib- IIIa NSCLC	30	Neoadjuvant	Nab- paclitaxel + CBDCA + atezolizumab +	MPR	MPR: 57% (IC 95% 37–75) Resection rate: 87% ORR: 63% DFS: 17.9 mo. (IC 95% 14.3–NA) mOS: NR (IC 95% 27.6–NR) Grade ≥ 3 TRAEs: neutropenia 50%, thrombocytopenia 7%, high LFTs 7%	Nab-paclitaxel is a valid and safe option for the chemotherapy backbone in IO-combined regimens
NCT03464734 (PEANUT trial), 2020	2	mUC	2nd or further line	70	Nab-paclitaxel + pembrolizumab	PFS	mPFS 5.9 mo. (IC 95% 3.1–11.5) ORR 38.6% (IC 95% 27.2%−51%), mOS: NE (IC 95% 9.5 mo-NE), 12-mo OS rate: 62.6% (IC 95% 46.8–74.9) Grade ≥ 3 TRAEs: neutropenia 8.6%, anemia 7.1%	Safe and effective combination
NCT02392637, 2019	2	BTC	66	1 st line	Nabpaclitaxel + cisplatin + gemcitabine	PFS	mPFS: 11.8 mo. (95% CI, 6.0–15.6) ORR: 45% DCR: 84% mOS: 19.2 mo. (95% CI, 13.2 mo—NA) Grade ≥ 3 TRAEs: 58% (most common neutropenia, 33%)	Despite the promising results, confirmatory trials should incorporate immunotherapy, in line with the new standard of care
NCT01812746, 2018	2	mCRPC	Post-ARSI progression	42	BIND-014 (PSMA-directed docetaxel-containing NP)	rPFS	≥ 50% PSA reduction: 30% (IC 95%, 18%−45%) rPFS: 9.9 mo. (IC 95%, 7.1–12.6 mo.) Grade ≥ 3 TRAEs: lymphopenia 12%, anemia 7%	Loss of PSMA-positive circulating tumor cells correlated with improved mOS (20.2 vs. 7.4 mo.; p = 0.06)
NCT01537536, 2016	2	HER2- negative breast cancer	Neoadjuvant	20	EndoTAG-1 (cationic liposomal PXT) + PXT followed by 3 cycles of FEC	MRI tumor volume reduction	Median MRI volume reduction: 6.36 cm^3^ (1.56 −40.87) at baseline vs 0.36 cm^3^ (0- 20.26) end of treatment, (p < 0.001) pCR: 33%	All the pCRs were in the TNBC population; 20% of the patients experienced hypersensitivity reaction and required permanent discontinuation of EndoTAG-1
NCT01159288, 2016	2	NSCLC	1 st line maintenance therapy	41	IFN-γ-Dex (tumor antigen loaded dendritic Cell exosomes)	4-month PFS rate ≥ 50%	4-month PFS rate: 32% (95% CI: 16–53) mPFS: 2.2 mo mOS: 15 mo Grade ≥ 3 TRAEs: 5% G3 AE, no G4 reported	Primary endpoint not reached. IFN-γ-Dex showed a manageable safety profile. NK cell function significantly improved in patients with PFS > 2.2 months (P < 0.05), with increased CD107a expression and cytokine production
NCT01426126, 2011	2	MIBC	Second line	37	Genexol PM (paclitaxel polymeric micelles)	ORR	ORR: 21% (95% CI: 7–34%) mPFS: 2.7 mo., (95% CI: 0.9–4.6 mo.) mOS: 6.5 mo, (95% CI: 5.0–8.0 mo.) Grade ≥ 3 TRAEs: PSP (sensory type 5.9%; motor type 8.8%) and infection (5.9%)	Genexol-PM was well tolerated and effective as second-line therapy
NCT02009332, 2021	1/2	NMIBC	BCG-refractory disease	13	Ph1: ABI-009 (nab sirolimus) 100–400 mg/week, 6w) Ph2: ABI-009 200 mg/week + gemcitabine (2000 mg/week, 6w	Ph1: DLT Ph2: pCR	DLT: not observed up to ABI-009 400 mg/week; MDD not reached pCR: 20% (1/5 patients)	ABI-009 exhibited minimal local toxicity and no systemic toxicity during the phase 1 trial
NCT03439462, 2020	1/2	mCRC	First-line	24	ABI-009 (nab-sirolimus) + FOLFOX + bevacizumab	RP2D	RP2D: 20 mg/m^2^ q2w. G3-4 TRAEs: 63% (most common: neutropenia 25%, thrombocytopenia 17%). Best response (n = 18): PR 39%, SD 56%, tumor shrinkage 89%	PTEN loss in 29% of pts; response rate higher in PTEN loss group (50%) vs PTEN WT (30%). Phase 2 ongoing
NCT02010567, 2019	1/2	Locally advanced rectal cancer	Neoadjuvant	32	CRLX101 (polimeric NP- camptothecin) + capecitabine + radiotherapy	Ph1: MTD Ph2: pCR	MTD: 15 mg/m^2^ weekly pCR rate: 19% overall, 33% at MTD Most common grade 3–4 AE: lymphopenia (25%)	Well tolerated, supporting further evaluation for the local control aligned with standard CRT regimens
NCT03190174, 2019	1/2	Sarcoma	2nd or further line	9	ABI-009 (nab-sirolimus) + nivolumab	MTD	MTD: not reached, 100 mg/m^2^ designated as Phase 2 dose mPFS: NE Grade ≥ 3 TRAEs: 11% hyperphosphatemia	Combination was feasible; phase 2 enrollment ongoing
NCT02043288, 2017	1/2	Advanced solid tumors	First-line and beyond	22	NC-6004 (polymeric NP-cisplatin) + gemcitabine	MTD, safety	MTD: 135 mg/m^2^ 55% had tumor shrinkage; ORR 15%; DCR 85% Reduced nephrotoxicity vs. cisplatin	Demonstrated prolonged systemic exposure with reduced toxicity. Phase 2 ongoing
NCT04573140, 2024	1	Glioblastoma MGMT WT	Post- surgery and CRT	3	RNA-LPA (multi-lamellar RNA–lipid particle aggregates)	Safety, immune activation, and preliminary efficacy	RNA-LPAs induced rapid cytokine release, immune cell mobilization, and expansion of glioma-specific T-cell responses. Tissue-confirmed pseudoprogression observed post-treatment	Tissue-confirmed pseudoprogression observed post-treatment indicates effective immune activation and infiltration
NCT04161755, 2023	1	PDAC	Adjuvant,	16	Autogene cevumeran (anionic mRNA–lipoplex, RNA-LPX) + atezolizumab + mFOLFIRINOX	Safety	Grade ≥ 3 TRAEs: 6% (fever and hypertension) Increased antigen-specific immune responses: 50% mRFS responders vs non responders: NE vs 13.4 mo (HR 0.08, CI 95%, 0.01–0.4; p = 0.003)	Phase 3 (IMCODE 003, BNT122) planned
NCT01946867, 2021	1	Locally advanced HNSCC	Elderly or frail patients ineligible for CRT	19	NBTXR3 (hafnium oxide nanoparticles) + IMRT	RP2D, safety, local response rate	RP2D: 22% of baseline tumor volume No DLTs observed CR in 56% of evaluable patients NBTXR3 remained in tumors throughout RT without leakage to healthy tissue	NBTXR3 demonstrated a favorable safety profile with promising local control in frail patients. Further evaluation in phase 2 warranted
NCT03164772, 2019	1/2	NSCLC	2nd or further line, intradermal	61	Intradermal CV9202 (protamine–mRNA complex) + durvalumab (arm A) or + durvalumab and tremelimumab (arm B)	Safety	TRAE: 56.5% and 55.9% SAE: 60.9% and 64.7% 24-mo PFS rate: 43.5% in arm A and 8.8% in arm B	
NCT02181075, 2017	1	HCC or liver metastases	Liver locoregional therapy	10	ThermoDox (lyso-thermosensitive liposomal doxorubicin) + focused ultrasound	twofold intratumoral doxorubicin concentration increase	Mean intratumoral drug concentration: threefold increase Mean concentration: 8.56 µg/g, Highest concentration: 21.8 μg/g (colon cancer) G4 TRAEs: neutropenia (50%)	Confirmed feasibility of non-invasive ultrasound-mediated targeted drug delivery. Supports further trials optimizing this approach
NCT02369198, 2017	1	Recurrent malignant pleural mesothelioma	Dose-escalation	27	TargomiRs (miR-16 mimic in EnGeneIC Dream Vector Minicells)	Safety, MTD	MTD: 5 × 10⁹ TargomiRs weekly. ORR: 5%, SD: 68% mOS: 200 days Most common AEs: infusion reactions, transient lymphopenia, and hypophosphatemia	First-in-human study of miRNA-loaded minicells. Well tolerated with early signs of antitumor activity. Further studies in combination with chemotherapy or ICIs needed
NCT01915524, 2016	1	NSCLC	2nd or further line, intradermal	26	Ph1: Intradermal CV9202 (protamine–mRNA complex) + local RT	Safety, immunogenicity	Ph1: Grade ≥ 3 TRAEs: 15.4% Increased antigen-specific immune responses: 84%	One PR, 46.2% SD. Further evaluation in combination with ICIs has been done in the phase 1/2
NCT02724176, 2016	Prospective cohort study	Papillary thyroid cancer	Lymph node mapping in central neck dissection	140	Carbon nanoparticles for sentinel lymph node detection	LN metastasis detection, parathyroid preservation	LN detection: 73% vs 54% (p = 0.017) Accidental parathyroid removal: 5 vs 14 (p = 0.046) Post-surgical hypocalcemia: 12 vs 23 patients (p = 0.033)	Carbon nanoparticles improved lymph node visualization and may protect parathyroid glands. Further validation needed
NCT02110563, 2016	1	Advanced solid tumors, multiple myeloma, lymphoma	Dose-escalation	19	DCR-MYC (siRNA in EnCore lipid nanoparticles)	Safety, pharmacokinetics, pharmacodynamics	Well tolerated across dose levels Most common TRAEs: fatigue (37%), nausea (26%), infusion reactions (16%) One patient had a sustained metabolic response for > 8 mo	First MYC-targeting siRNA therapy in clinical trials. Demonstrated early signs of metabolic response and tumor shrinkage. Further evaluation needed
NCT00346229, NCT00826085, 2014	1	Locoregionally recurrent breast cancer	2nd and further line	29	LTLD + mild local hyperthermia	MTD, safety, local response rate	MTD: 50 mg/m^2^ Local response rate: 48% (17% CR, 31% PR) TRAE G ≥ 3–4: neutropenia (24%), leukopenia (14%)	Two phase 1 trials confirmed feasibility and promising local control in heavily pretreated patients. Further evaluation in earlier-stage disease warranted

### FDA-approved drugs

Doxil (liposomal doxorubicin), the first FDA-approved nanomedicine, was specifically designed to treat AIDS-related Kaposi sarcoma [94, 95], with expanded approvals for ovarian cancer [96, 97] and multiple myeloma [98]. In the breast cancer setting, Doxil remains an off-label use, while in Europe a non-PEGylated formulation has been approved by the EMA (i.e., Myocet) [99]. Abraxane (nab-paclitaxel), an albumin-bound paclitaxel, offers a solvent-free formulation for treating breast cancer [100, 101], non-small cell lung cancer (NSCLC) [102], and pancreatic cancer [103] that reduces hypersensitivity reactions associated with traditional solvents and provides better tumor penetration [104].

Another liposomal formulation is Onivyde (nanoliposomal irinotecan, NAL-IRI), which was first approved for the treatment of refractory metastatic pancreatic cancer. This formulation provided a sustained intratumoral release of irinotecan, thus prolonging the drug’s activity and improving patient outcomes [105]. Recently, the FDA approved Onivyde for the first-line treatment setting as part of the NALIRIFOX combination, following the statistically significant clinical benefits demonstrated by the phase III NAPOLI-3 trial (overall survival: hazard ratio 0.84, *p* = 0.04; progression-free survival: hazard ratio 0.70, *p* = 0.0001) [106].

DepoCyt (cytarabine liposome) is indicated for lymphomatous meningitis. Its liposomal structure allows cytarabine to reach the cerebrospinal fluid over extended periods, ensuring sustained therapeutic levels and enhancing patient convenience [107, 108]. VyxEOS (daunorubicin and cytarabine liposome) was specifically developed for newly diagnosed therapy-related AML or AML with myelodysplasia-related changes [25]. The liposomal co-encapsulation of daunorubicin and cytarabine facilitates a synergistic effect, optimizing the ratio of the drugs at the tumor site and achieving higher remission rates in AML patients [25].

As shown in Table 1, many other drugs have obtained regulatory approval, including those aimed at the supportive care setting. Neulasta (pegfilgrastim) and Emend (aprepitant) are key examples. Neulasta, a pegylated granulocyte-colony stimulating factor, reduces the incidence of infection in patients receiving myelosuppressive chemotherapy by stimulating neutrophil production [109–111]. Emend, a neurokinin-1 (NK1) receptor antagonist, is crucial in preventing chemotherapy-induced nausea and vomiting, thereby significantly enhancing the quality of life for patients undergoing cancer treatment [112].

### Selected phase I-III investigational trials

The landscape of clinical trials in cancer nanomedicine is extensive. The results of the completed trials are detailed in Table 2**.**

Only a few nanomedicine-based drugs have advanced to phase 3 trials. Among them, nab-paclitaxel demonstrated superior efficacy over conventional paclitaxel as a neoadjuvant treatment for early-stage breast cancer in two randomized trials, registering the most benefit in triple-negative breast cancer (TNBC) [113, 114]. Conversely, two other studies evaluating NK105 (micellar paclitaxel) versus conventional paclitaxel as first-line therapy in metastatic breast cancer and NKTR-102 (irinotecan pegol) versus a physician’s choice regimen in pretreated metastatic breast cancer failed to meet their primary endpoints. However, both nanotechnology-based formulations demonstrated an improved toxicity profile, with NK105 significantly reducing peripheral sensory neuropathy (p < 0.0001) and NKTR-102 showing fewer grade ≥ 3 adverse events (p < 0.0001) compared with their control groups [115, 116]. The HEAT study [117] assessed thermosensitive liposomal doxorubicin plus radiofrequency ablation (RFA) in unresectable hepatocellular carcinoma, showing no benefit in progression-free or overall survival. A post-hoc analysis revealed an overall survival advantage only in solitary lesions treated with an RFA dwell time ≥ 45 min (p < 0.05), suggesting a proportionality between the extent of RFA-mediated heat and drug release [117].

Among phase 2 trials, nab-paclitaxel has been explored in multiple unapproved disease settings. In biliary tract cancers it was tested as part of a triplet regimen with cisplatin and gemcitabine in two positive single-arm phase 2 trials, one conducted in the first-line metastatic setting [118] and the other in the neoadjuvant setting for high-risk resectable disease (NeoGAP trial). The NeoGAP trial [119] reported promising results (Table 2), supporting further evaluation with an active comparator arm in the ongoing phase 2/3 PURITY trial (NCT06037980). Similarly, nab-paclitaxel demonstrated clinical activity in head and neck squamous cell carcinoma (HNSCC) as induction therapy for locally advanced disease [120] and in the second-line metastatic setting in combination with nivolumab, where phase 2 accrual is ongoing (NCT04831320).

Other taxane-loaded nanoformulations have been explored in phase 2 trials. Prostate-specific membrane antigen-targeted docetaxel nanoparticles (BIND-014) improved clinical outcomes in a single-arm trial in patients with pretreated metastatic castration-resistant prostate cancer [121]. Polymeric micelle paclitaxel (Genexol-PM) has been tested in urothelial carcinoma as second-line therapy following gemcitabine-cisplatin in a single-arm study, demonstrating good clinical activity and a manageable toxicity profile [122]. Cationic liposomal paclitaxel (EndoTAG-1) was investigated in HER2-negative breast cancer in the neoadjuvant setting in combination with paclitaxel followed by the FEC (fluorouracil, epirubicin, and cyclophosphamide) chemotherapy regimen. Notably, pCR was a secondary endpoint, observed in 33% of cases, all of whom had TNBC [123].

Beyond taxanes, liposomal irinotecan (LY01610) has been evaluated in relapsed small cell lung cancer, where a phase 2 single-arm trial identified the 80 mg/m^2^ regimen as the most effective, yielding a duration of response of 6.9 months (95% CI: 2.5–9.9) and a manageable safety profile [124]. In an ongoing phase 3 trial (NCT05561036), LY01610 is being compared to an active control arm in patients who progressed after first-line chemo-immunotherapy.

Albumin-bound sirolimus (nab-sirolimus), an mTOR inhibitor, has been investigated across multiple malignancies. In high-grade glioma and glioblastoma, a phase 2 trial (NCT03463265) explored its combination with temozolomide, bevacizumab, lomustine, or marizomib, as well as with radiotherapy plus temozolomide in the first-line setting, with overall limited efficacy. In metastatic colorectal cancer, a phase 1/2 study assessed nab-sirolimus plus mFOLFOX and bevacizumab. The study reported dose-dependent hematologic toxicities but also tumor shrinkage in 89% of evaluable patients, with promising responses in particular in tumors harboring PTEN loss (NCT03439462).

In two phase 1/2 trials, polymeric nanoparticles demonstrated clinical activity in distinct tumor types. CRLX101, a nanoparticle formulation loaded with camptothecin, was evaluated in combination with capecitabine and radiotherapy for neoadjuvant treatment of locally advanced rectal cancer, achieving effective local disease control without compromising surgical radicality [125]. Similarly, NC-6004, a cisplatin-containing nanoparticle, was assessed in advanced solid tumors and demonstrated prolonged systemic exposure, reduced nephrotoxicity compared with historical cisplatin cohorts, and a disease control rate of 85% [126].

Theranostic nanoparticles have been investigated for both targeted cancer treatment and surgical guidance. In a phase 2 trial, gold nanoshell-directed photothermal ablation was explored as a focal therapy for localized prostate cancer, achieving tumor control in 73% of patients at 12 months with significant prostate-specific antigen reduction (p < 0.0001) and no grade 3–4 adverse events [127].

In papillary thyroid cancer, a prospective cohort study evaluated the use of carbon nanoparticles for sentinel lymph node detection, demonstrating improved identification of metastatic lymph nodes (p = 0.017) and reduced accidental parathyroid removal (p = 0.046) during central neck dissection [128].

In the era of immune checkpoint inhibitors, alternative immunotherapy-related strategies leveraging nanotechnology have been explored. For instance, a phase 2 trial (2016) investigated dendritic cell-derived exosomes as maintenance therapy in NSCLC but failed to meet its primary endpoint despite improving natural killer cell function. [129] In contrast, nanoparticle-based vectors have significantly advanced the clinical application of mRNA-based cancer vaccines by enhancing nucleic acid stability, targeted delivery, and immune activation. Four main nanocarrier platforms have been studied: protamine–mRNA complexes, anionic RNA–lipoplexes, multi-lamellar RNA–lipid particle aggregates, and RNA–lipid nanoparticles.

Protamine–mRNA complexes, such as CV9202, were tested in NSCLC and administered intradermally in combination with local radiotherapy in a phase 1 trial (NCT01915524). The subsequent phase 1/2 study further optimized the regimen by incorporating durvalumab and tremelimumab [130].

Anionic RNA–lipoplexes, including autogene cevumeran, were administered intravenously in combination with atezolizumab and mFOLFIRINOX in the adjuvant setting for pancreatic ductal adenocarcinoma. This approach employed selective spleen localization to enhance immune priming and induced robust neoantigen-specific T-cell responses in 50% of patients. Responders exhibited a significantly prolonged recurrence-free survival compared to non-responders (median not reached vs. 13.4 months, p = 0.003). These promising results have led to the initiation of a global phase 3 randomized trial (IMCODE 003, BNT122) [131].

Intravenous administration of multi-lamellar RNA–lipid particle aggregates, designed to increase payload capacity and systemic immune activation, are under investigation in glioblastoma [132].

Finally, RNA–LNPs, exemplified by V940 (mRNA-4157), represent the most successful nanocarrier platform, demonstrating unprecedented efficacy as adjuvant treatment in stage III-IV melanoma (Table 2). Intramuscular administration in combination with pembrolizumab has been successfully tested in the positive KEYNOTE-942 trial, with significant clinical improvements in terms of disease-free survival (HR 0.561; 95% CI, 0.309–1.017; p = 0.053) and distant metastases-free survival (HR 0.347; 95% CI, 0.145–0.828; p = 0,013) over pembrolizumab alone [28], supporting the ongoing phase 3 trial (NCT05933577).

RNA interference (RNAi)-based therapeutics have been tested in first-in-human phase 1 trials, leveraging the enhanced delivery capacity of nanocarriers. DCR-MYC, a synthetic siRNA targeting MYC encapsulated in EnCore lipid nanoparticles, was evaluated in patients with advanced solid tumors, multiple myeloma, and lymphoma, demonstrating a favorable safety profile (NCT02110563). TargomiRs, a microRNA(miR)−16 mimic encapsulated in EnGeneIC Dream Vector minicells targeting EGFR, were evaluated in recurrent malignant pleural mesothelioma in a first-in-human phase 1 trial. As a tumor-suppressor miRNA, miR-16 restored post-transcriptional regulation of oncogenic pathways, leading to early signs of disease stabilization, with 68% of patients achieving stable disease, 5% achieving a partial response, and a median overall survival duration of 200 days [133].

## Preclinical studies with nanotechnology-based drugs

Preclinical studies are crucial in evaluating the efficacy, safety, and targeting efficiency of nanotechnology-based drug delivery systems before they are advanced to clinical trials. Various nanoparticle formulations have been developed and tested in vitro and in vivo and have demonstrated enhanced drug delivery, reduced toxicity, and improved therapeutic outcomes in cancer and other diseases. Table 3 provides a comprehensive overview of preclinical studies on nanotechnology-based drugs across different cancer types.

**Table 3:** Selected preclinical studies with nanotechnology in cancer treatment: applications, nanoparticles, and delivery systems

Investigator, Year	Cancer Type	Nanoparticles Used	Nanoparticle Drug Delivery Systems	Findings
Li et al. 2013 [137]	Tumor model (KB cells)	Folic acid-targeted Fe3O4 NPs	PEI-mediated synthesis and PEGylation	• PEI-coated Fe3O4 NPs are stable and water-dispersible; cytocompatible and hemocompatible • Successfully targeted KB cancer cells (FA receptors) in vitro and in vivo MR imaging of xenografted tumors
Li et al., 2014 [214]	Brain metastases of breast cancer	Poly(methacrylic acid)–polysorbate 80-grafted-starch nanoparticles	Multifunctional nanotheranostic system delivering doxorubicin (Dox) and imaging agents	• Confirmed extravasation of gadolinium and dye-loaded nanoparticles across the BBB in healthy mice • Targetability of Dox-loaded nanoparticles to brain metastases assessed via imaging • Coexistence of nanoparticles and Dox in tumors confirmed histologically • Induced apoptosis in cancer cells 24 h post-injection while sparing normal cells • Significantly inhibited tumor growth compared to free Dox at the same dose
Wei et al. [215]., 2014	Drug-resistant tumors expressing CD44 receptors	Cholesteryl-modified hyaluronic acid (CHA) nanogels	• CHA-drug conjugates with hydrophobic cores, loaded with etoposide, salinomycin, or curcumin	• Nanogels (20–40 nm) with up to 20% drug load • Sustained drug release via hydrolysis of ester linkage • 2–7 times higher cytotoxicity in CD44-expressing drug-resistant breast and pancreatic cancer cells compared to free drugs • Efficient internalization via CD44-mediated endocytosis and membrane interaction • Enhanced penetration and cytotoxicity in multicellular cancer spheroids
Gao et al., 2014 [138]	HCC	Polymer-lipid hybrid nanoparticles (PLNPs)	• Anti-EGFR antibody conjugated PLNPs loaded with adriamycin	• Improved cytotoxicity, targeted delivery, and tumor suppression in HCC
Goe et al., 2014 [143]	Glioblastoma	VEGF121-conjugated mesoporous silica nanoparticles	• Targeted PET imaging and sunitinib delivery	• Efficient drug delivery and enhanced imaging of glioblastoma tumors
Clark and Davis, 2015 [139]	Brain cancer	80-nm gold nanoparticles with transferrin (Tf) or anti-TfR antibodies	• Acid-cleavable linkage between Tf/Abs and nanoparticles for receptor-mediated transcytosis (RMT) across the BBB	• Tf-containing nanoparticles with cleavable linkages showed increased brain uptake compared to non-cleavable ones • Antibody-based nanoparticles had lower uptake due to endothelium retention
Obaid et al. 2015 [144]	CRC adenocarcinoma (HT-29 cells) and breast adenocarcinoma (SK-BR-3 cells)	Water-soluble gold nanoparticles (AuNPs) conjugated with zinc phthalocyanine (C11Pc), PEG, and either jacalin (a lectin) or anti-HER-2 antibodies	• AuNPs for enhanced delivery of the photosensitizer • C11Pc as a photosensitizer for photodynamic therapy (PDT) • Jacalin to target the Thomsen-Friedenreich (T) antigen • Anti-HER-2 antibodies to target HER-2 receptors	• Jacalin- and antibody-conjugated nanoparticles exhibited similar singlet oxygen generation and phototoxicity levels • Targeted nanoparticles had significantly higher phototoxicity than non-conjugated nanoparticles • Both conjugates are localized in lysosomes, indicating receptor-mediated endocytosis • Targeting the T antigen with jacalin was as effective as targeting HER-2 with antibodies in PDT
Jørgensen et al., 2016 [145]	Human tumor xenografts in mice	Near-infrared resonant silica-gold nanoshells (AuNSs), solid gold nanoparticles (AuNPs)	• Single particle and PET-based platform	• AuNSs demonstrated superior heat generation and photothermal efficiency compared to AuNPs, both in vitro and in vivo • PET imaging (using 18 F-FDG) successfully monitored early treatment response, validating the use of the platform for benchmarking plasmonic nanoparticles in cancer therapy
Kim MS et al., 2016 [147]	MDR cancer	Exosome-encapsulated paclitaxel (exoPTX)	• Natural exosome-based drug delivery	• Increased cytotoxicity and improved targeting in MDR cancer models
Sambade et al., 2016 [140]	NSCLC (brain metastases)	PRINT® PLGA nanoparticles of docetaxel and acid-labile C2-dimethyl-Si-docetaxel	• Intravenous injection of nanoparticle formulations of docetaxel and C2-dimethyl-Si-docetaxel (acid-labile)	• Intracranial tumor concentrations of PRINT-docetaxel were 13-fold higher and PRINT-C2-docetaxel sevenfold higher than small molecule (SM)-docetaxel • C2-docetaxel conversion to docetaxel was threefold higher in tumor tissues compared to non-tumor tissues • PRINT-C2-docetaxel increased median survival by 35% with reduced toxicity compared to other treatments
Yao et al., 2016 [216]	Breast cancer (4 T1 cells as model system)	Graphene Quantum Dots (GQDs)-Capped Magnetic Mesoporous Silica Nanoparticles (MMSN)	• MMSN nanoparticles loaded with doxorubicin (DOX) for chemotherapy, magnetic hyperthermia, and photothermal therapy	• MMSN/GQDs nanoparticles (100 nm) efficiently loaded DOX and triggered its release in a low pH environment • MMSN/GQDs generated heat under an alternating magnetic field or near-infrared irradiation, achieving hyperthermia temperature • Combined chemo-magnetic hyperthermia or chemo-photothermal therapy with DOX-loaded MMSN/GQDs significantly enhanced the therapeutic efficiency, killing more cancer cells compared to individual therapies
Xu et al., 2016 [149]	Breast cancer (MCF-7 cells)	Amine functionalized hydroxyapatite (NHAP) nanoparticles	• NHAP nanoparticles combined with anti-angiogenesis (ANG) plasmid for gene therapy	• ANG/NHAP nanoparticles were around 50 nm in diameter and showed effective plasmid condensation • Cellular assays confirmed high transfection efficiency, low cytotoxicity, and significant anti-angiogenesis activity • ANG/NHAP nanoparticles are suggested as a safe and effective drug delivery system for potential breast cancer gene therapy
Wen L et al., 2016 [157]	Deep-seated liver tumors	Single-wall carbon nanotubes	• Microwave-pumped thermoacoustic tumor therapy	• Selective targeting and destruction of tumor mitochondria; effective in deep-seated tumors
Prava and Raj 2016 [156]	Not specified (in vitro cytotoxicity tested)	Iron oxide nanoparticles (Fe3O4) coated with β-cyclodextrin (β-CD), PEG, and PEI, loaded with 5-fluorouracil (5-FU)	• Fe3O4 as the core for potential magnetic targeting • β-CD, PEG, and PEI as coating agents for stability and drug loading • 5-FU as the anticancer drug	• 5-FU-loaded nanoparticles exhibited toxicity towards cancer cells but not normal cells • Released 5-FU more rapidly and at higher levels at pH 6.8 compared to acidic pH 1.2
Wadajkar et al., 2017 [148]	GBM	Poly(lactic-co-glycolic acid) (PLGA) and PLGA-polyethylene glycol (PLGA-PEG)	• DART therapeutics with decreased non-specific adhesivity and receptor targeting	• Minimized non-specific binding in the brain microenvironment • Enhanced binding to Fn14 receptor • Preserved nanoparticle diffusivity in brain tissue • Increased cellular uptake in tumor cells • Longer retention in orthotopic tumors compared to non-targeted versions
Gu L et al., 2017 [217]	NSCLC with KRAS mutation and p53 loss	Layer-by-layer nanoparticles	• Core liposomes encapsulating cisplatin, layered with polyelectrolytes including siKRAS and miR-34a, and an outer hyaluronic acid layer for targeting	• Enhanced toxicity against lung adenocarcinoma cells • Preferential uptake in lungs of tumor-bearing mice • Prolonged survival in treated mice • Potential for clinical application in NSCLC therapy
Penon et al. 2017 [218]	Human breast cancer (SK-BR-3 cells)	Water-soluble porphyrin-gold nanoparticle conjugates with anti-erbB2 antibody	• Gold nanoparticles (AuNPs) for enhanced delivery • Porphyrin as a photosensitizer for PDT • Anti-erbB2 antibody for targeted delivery to erbB2-positive cancer cells	• Successful synthesis of water-soluble antibody-porphyrin-AuNP conjugates • Monophasic synthesis method produced nanoparticles with higher singlet oxygen generation • Antibody-porphyrin-AuNP conjugates effectively targeted and killed erbB2-positive breast cancer cells via PDT
Amreddy et al., 2018 [219]	Lung cancer (H1299 cells)	Folate receptor-targeted polyamidoamine dendrimer nanoparticles (Den-based)	• Folic acid (FA)-conjugated Den nanoparticles for co-delivery of HuR siRNA and cis-diamine platinum (CDDP) to folate receptor-α (FRA)-overexpressing lung cancer cells	• FRA-targeted NP showed significantly higher therapeutic efficacy in co-delivery of HuR siRNA and CDDP than individual therapies • FRA-targeted NP exhibited enhanced cytotoxicity compared to non-targeted NP • The system showed negligible toxicity towards normal lung fibroblasts (MRC9 cells)
Sun Y et al., 2018 [190]	NSCLC	Cysteine-modified iron-platinum (FePt-Cys) nanoparticles	• FePt-Cys NPs inducing reactive oxygen species (ROS) generation	• Induced ROS burst leading to apoptosis in NSCLC cells • Suppressed antioxidant protein expression • Inhibited migration and invasion of H1975 and A549 cells • Decreased MMP-2/9 expression and enhanced cellular attachment • Enhanced effects of cisplatin and radiation therapy by activating caspase system and impairing DNA damage repair • Demonstrated good solubility, stability, biocompatibility, and safety in vivo
Moghimipour et al., 2018 [135]	CRC	Folic acid-modified liposomes	Targeted delivery of 5-fluorouracil (5-FU)	• Enhanced cytotoxicity, targeted drug delivery, and reduced tumor volume compared to free 5-FU
Kim JS et al., 2018 [220]	GBM	Dual-targeting immunoliposomes	Liposomes conjugated with angiopep-2 and anti-CD133 monoclonal antibody, encapsulating temozolomide (TMZ)	• Dual-targeting liposomes effectively crossed the blood–brain barrier and targeted glioblastoma stem cells (GSCs) • In vitro*,* Dual-LP-TMZ increased cytotoxicity against U87MG GSCs by 425-fold compared to free TMZ • In vivo, treatment with Dual-LP-TMZ significantly reduced tumor size and prolonged survival in orthotopic brain tumor mouse models
Abazari et al. 2018 [155]	Breast cancer (MCF-7)	Bio-metal–organic framework (Bio-MOF) coated with chitosan (CS)	• pH-responsive, target-selective delivery system for doxorubicin (DOX) • Drug release assessed at different pH levels (PBS, pH 7.4 and 6.8)	• Slow, continuous release profile at pH 7.4, and significant release (93%) at pH 6.8 • Enhanced cellular uptake and apoptosis in MCF-7 cells • Biocompatible with high drug loading capacity (21.7% at pH 7.4)
Lang FM et al., 2018 [154]	Gliomas	Exosomes derived from mesenchymal stem cells (MSCs)	• MSCs engineered to overexpress miR-124a, producing exosomes (Exo-miR124) containing high levels of miR-124a	• miR-124a identified as a potent antiglioma microRNA • Exo-miR124 significantly reduced viability and clonogenicity of glioma stem cells (GSCs) in vitro • Systemic delivery of Exo-miR124 in mice with intracranial GSCs led to long-term survival in 50% of treated animals • Mechanistic studies showed miR-124a silences FOXA2, causing aberrant lipid accumulation in GSCs
Kakali De et al., 2021 [134]	Prostate and breast cancer (PC3 and SKBR3)	Decapeptide-modified solid lipid nanoparticles (SLNs)	• Targeted delivery of doxorubicin using LHRH-receptor binding SLNs	• Enhanced targeting and cytotoxicity in prostate cancer cells; improved apoptosis and reduced side effects
Liu et al., 2019 [221]	Orthotopic CRC	Silicasomes	• Silica-based nanoparticles encapsulating irinotecan	• Enhanced therapeutic efficacy in orthotopic colon cancer models • Reduced systemic toxicity compared to free irinotecan • Improved drug delivery and retention at tumor sites • Potential for clinical translation in colon cancer treatment
Ebadi et al., 2019 [222]	Liver cancer (HepG2 cells)	Iron oxide nanoparticles (Fe3O4) coated with PEG and co-coated with 5-fluorouracil/Mg/Al-LDH or 5-fluorouracil/Zn/Al-LDH	• Fe3O4 as the core for magnetic properties; PEG as a stabilizing agent; LDH as the drug carrier; 5-fluorouracil (5-FU) as the anticancer drug	• Demonstrated enhanced anticancer activity against HepG2 cells compared to free 5-FU • Exhibited reduced toxicity towards normal fibroblast 3 T3 cells
Kadiyala O et al., 2019 [223]	GBM	HDL-mimicking nanodiscs	• Nanodiscs loaded with doxorubicin (DOX) and Toll-like receptor 9 (TLR9) agonist CpG	• Nanodiscs effectively delivered DOX and CpG to GBM tumors • Combination therapy induced immunogenic cell death • Enhanced activation of dendritic cells and T cells • Significant inhibition of tumor growth and prolonged survival in mouse models
Hu M et al., 2019 [158]	Liver metastasis from colorectal, pancreatic, and breast cancers	Aminoethyl anisamide-conjugated lipid-calcium-phosphate (LCP) nanoparticles	• LCP nanoparticles delivering plasmid DNA encoding relaxin (pRLN)	• Targeted delivery to metastatic tumor cells and activated hepatic stellate cells • Reversed stromal microenvironment, inhibiting metastatic progression • Prolonged survival in mouse models • Reactivated intra-metastasis immune milieu • Synergistic effect with PD-L1 blockade immunotherapy, enhancing anti-metastatic efficacy
Chen et al., 2019 [141]	Pancreatic cancer	TR peptide-modified liposomes	• Co-delivery of paclitaxel and hydroxychloroquine	• Synergistic anti-cancer and anti-stromal effects in pancreatic ductal adenocarcinoma
Zhang et al., 2019 [224]	ATC	131I-labeled anti-VEGFR2 mesoporous silica nanoparticles	• 131I-labeled anti-VEGFR2 mesoporous silica nanoparticles	• Enhanced targeting, increased tumor retention, and prolonged survival in mouse models of ATC
Ebadi et al., 2020 [225]	Liver cancer (HepG2 cells)	FeNPs coated with PVA/LDH or PEG/LDH and loaded with sorafenib	• FeNPs as the core for magnetic properties PVA or PEG as coating agents • Magnesium–aluminum layered double hydroxide (MLDH) as the drug carrier • Sorafenib as the anticancer drug	• Approximately 85% of sorafenib was released from the nanoparticles within 72 h, following pseudo-second-order kinetics • The coated nanoparticles loaded with sorafenib demonstrated anticancer activity against HepG2 cells • Lower toxicity was observed in fibroblast-type 3 T3 cells compared to the pure drug
Tsakiris et al., 2020 [226]	CRC	SN38 and salinomycin nanoparticles	• Solid lipid nanocapsules	• Tested on colorectal cancer cell lines and in vivo murine models. Targeted proliferating cancer cells (via SN38) and therapy-resistant cancer stem cells (via salinomycin), improving survival and reducing systemic toxicity
Khan and Sahu, 2020 [227]	Breast cancer (MCF-7 cells)	Polyethylene glycol-diamine functionalized mesoporous SPION	• SPIONs prepared via a solvothermal method • Folic acid (FA) attached for targeting via carbodiimide chemistry	• High drug-loading efficiency (~ 96%) due to mesoporous structure • NPs achieved hyperthermic temperature of 43 °C within 223 s under alternating magnetic field • Non-appreciable toxicity in MCF-7 cells until loaded with doxorubicin
Asghar et al., 2020 [228]	Tumor cells (RAW 264.7 cells)	Thermoresponsive polymer-coated, superparamagnetic Fe3O4 embedded hollow mesoporous silica nanoparticles (HmSiO2-F-P(NIPAM-MAm))	• HmSiO2-F-P(NIPAM-MAm)-Dox (doxorubicin-loaded)	• Synthesis and characterization of nanocarriers with high loading capacity (95% encapsulation efficiency) • Biocompatibility confirmed • Significant anticancer activity against HeLa cells • pH and temperature-dependent drug release profile
Ou et al., 2020 [229]	OSCC	Graphene oxide-polyethylenimine	• miRNA inhibitor delivery for gene therapy	• Reduced tumor growth, increased apoptosis, and suppression of metastasis in OSCC
Chowdhury et al., 2020 [230]	Her-2 + breast cancer (MCF-7 and SKBR-3 cells)	Aptamer-labeled liposomes loaded with doxorubicin (DOX)	• Liposomes composed of various saturated and unsaturated lipids (HSPC, DPPC, POPC, DOPC) • Aptamer A6 for targeted delivery to HER2 + cells	• Liposomal formulations had small particle sizes (< 200 nm) and high drug encapsulation efficiency (≈ 88 ± 5%) • Aptamer-labeled liposomes (F5) demonstrated over 60% increased uptake in HER2 + cells compared to non-targeted liposomes • F5 achieved approximately 1.79-fold higher DOX uptake in HER2 + cells than in HER2- cells
Crous and Abrahamse et al., 2020 [231]	Lung cancer stem cells	Gold nanoparticles (AuNPs) conjugated with photosensitizer (AlPcS4 Cl) and antibody (Ab)	• AuNPs for drug delivery and retention • Antibody for targeted delivery to lung CSCs • AlPcS4 Cl as a photosensitizer for PDT	• Successful conjugation of the nanobioconjugate (NBC) confirmed • NBC localized in integral organelles of lung CSCs • AlPcS4 Cl-AuNP-Ab induced significant cell toxicity and death compared to free AlPcS4 Cl • Enhanced PDT effect observed with the NBC, leading to significant lung CSC destruction
Yin J. et al., 2021 [150]	General cancer immunotherapy	Polyethylenimine-functionalized graphene oxide hydrogel	• In situ transforming RNA nanovaccine delivery for immunotherapy	• Improved tumor antigen presentation, increased CD8 + T-cell activation, long-term antigen-specific immunity, and efficient prevention of metastasis
Nunes et al., 2021 [232]	CRC cells	Folate-coated pH-sensitive liposomes	• Encapsulation of irinotecan for controlled release	• Improved antitumor activity with reduced systemic toxicity in murine colorectal cancer model
Mulens-Arias V. et al., 2021 [177]	Colon peritoneal metastasis	Gold nanoparticles (AuNPs) conjugated with fluorouracil (5-FU)	• Systemic administration of 5-FU-AuNPs followed by near-infrared (NIR) laser irradiation to induce mild hyperthermia	• Selective accumulation of 5-FU-AuNPs in tumor tissues • NIR laser irradiation induced mild hyperthermia (40–42 °C) in tumor sites • Combined treatment enhanced antitumor efficacy compared to chemotherapy alone • Increased infiltration of immune cells, including cytotoxic T lymphocytes, into tumor microenvironment • Induction of immunogenic cell death markers, such as calreticulin exposure and HMGB1 release • Reduced tumor growth and prolonged survival in mouse models
Luiz et al., 2022 [233]	Breast cancer	Folic acid-modified curcumin-loaded liposomes	• Targeted drug delivery to folate receptors	• Enhanced cytotoxicity, increased cellular uptake, and improved penetration in 3D tumor models
Honarvari et al. 2022 [234]	Breast cancer	Folate-targeted curcumin-loaded biosomes	• Site-specific delivery to breast cancer cells	• Improved curcumin efficacy in breast cancer; reduced side effects
Tunç C.Ü et al., 2022 [134]	TNBC & MCF7	AuNPs	siRNA-functionalized AuNPs with intercalated doxorubicin (Dox)	• Efficient co-delivery of Bcl-2 siRNA and Dox • Significant downregulation of Bcl-2 gene expression (40% reduction) • Increased apoptosis (~ 35% vs. 24% with free Dox) • Enhanced inhibition of cancer cell proliferation (70–82% reduction in TNBC cells) • Decreased cancer cell migration and colony formation • Biocompatible and scalable approach with no need for cationic polymers
Radzi MRM, 2022 [235]	Breast cancer	Oxidized multiwalled carbon nanotubes (O-MWCNTs)	• O-MWCNTs administered intravenously, followed by near-infrared (NIR) laser irradiation to induce hyperthermia	• O-MWCNTs demonstrated efficient photothermal conversion upon NIR laser exposure • In vivo studies showed significant tumor growth inhibition in treated mice • Histopathological analysis revealed increased tumor cell apoptosis and necrosis • Minimal adverse effects observed in vital organs, indicating biocompatibility of O-MWCNTs
Mkhobongo et al. 2023 [236]	Metastatic melanoma stem cells (CD133 + A375 cell line)	Aluminum phthalocyanine conjugated to gold nanoparticles (AlPcS4 Cl-AuNP)	• Gold nanoparticles (AuNPs) for enhanced delivery of the photosensitizer • AlPcS4 Cl as a photosensitizer for photodynamic therapy (PDT)	• The AlPcS₄Cl-AuNP conjugate mediated PDT that promoted apoptotic cell death in melanoma stem cells • Increased expression of p53 and caspase-3 indicated apoptosis • Enhanced PDT effects were observed with the AlPcS₄Cl-AuNP conjugate compared to AlPcS₄Cl alone
Ilangovan SS, Mahanty et al., 2023 [237]	Breast cancer (MCF-7 cells), liver cancer (HepG2 cells), lung cancer (NCIH460 cells)	Superparamagnetic iron oxide nanoparticles (SPIONs) conjugated with β-sitosterol (BS) and coated with PEG and/or PNIPAM	• SPIONs, PEG, and PNIPAM as modifiers to enhance BS delivery • Various conjugates: BS-S, BS-SP, BS-SPP	• Increased size, stability, and monodispersity observed in the order of BS-S, BS-SP, BS-SPP • Highest drug encapsulation efficiency in BS-SPP (82.5%) • Sustained drug release in BS-SP (82.6%) and BS-SPP (83%) • IC50 values indicate highest inhibition towards NCIH 460 cells (164 µg/mL) Potential for targeted therapy against EGFR and MET receptor-expressing cancer cells
Taghikhani et al., 2024 [238]	Breast cancer (MCF-7)	Magnetic layered double hydroxides/Cu metal–organic framework-chitosan crosslinked к-carrageenan	pH-sensitive biocompatible hydrogel nanoparticles (LDH-Fe3O4/Cu MOF-DOX-CS@CAR) for controlled doxorubicin delivery	• High encapsulation efficiency (96.1%) and drug loading capacity (9.6%) • Controlled release: 60.3% at pH 5.5 vs. 22.6% at pH 7.4 after 72 h • Enhanced cytotoxicity toward MCF-7 cells with biocompatibility for L929 cells • Exhibited excellent antioxidant activity (71.81%) and blood compatibility (< 5%)
Simelane and Abrahams, 2024 [239]	CRC (Caco-2 cells in 3D MCTS)	PEGylated gold nanoparticles (PEG-AuNPs) conjugated with photosensitizer (ZnPcS₄) and anti-guanylate cyclase monoclonal antibodies (mAb)	• PEG-AuNPs for enhanced delivery of the photosensitizer • Anti-guanylate cyclase mAb for targeted delivery to CRC cells • ZnPcS₄ as a photosensitizer for PDT	• Enhanced anticancer effects observed in Caco-2 3D MCTS after PDT using the BNC nanoconjugate • Targeted BNC nanoconjugates improved PDT efficacy in a 3D tumor model
Ji D, et al., 2024 [240]	Lung cancer	Chimeric antigenic peptide influenza virus (CAP-Flu)	• Attenuated influenza A virus conjugated with CpG and covalently linked to tumor antigens	• Intranasal administration led to increased immune cell infiltration in tumors • Enhanced antigen uptake by dendritic cells • Specific immune cell response with increased tumor-infiltrating lymphocytes • Engineered virus expressing anti-PD-L1 nanobodies further enhanced tumor regression and prolonged survival in mouse models

### Emerging targeted strategies for peptide and liposomal drug delivery

A recent key strategy to reduce payload off-target toxicity is the functionalization of nanoparticles using ligands targeting overexpressed cancer receptors. For instance, PEGylated SLNs conjugated with an LHRH analog were tested on three cell lines: LNCaP prostate cancer cells with high LHRH receptor expression, MCF-7 breast cancer cells with low receptor expression, and normal renal cells. The modified SLNs exhibited higher uptake, cytotoxicity, and apoptosis induction in LNCaP cells compared with both MCF-7 and normal cells, suggesting that this strategy has high cancer selectivity [134]. Folic acid (FA)-PEG-liposomes encapsulating 5-fluorouracil demonstrated enhanced cellular uptake, increased ROS production, and lower IC_50_ values in colorectal cancer cell lines while maintaining excellent blood biocompatibility. In vivo, they significantly enhanced cytotoxicity and achieved tumor volume reduction [135, 136]. Folic acid-targeted magnetic iron oxide nanoparticles (Fe₃O₄ NPs) showed stability, water dispersibility, and successful targeting of cancer cells expressing folate receptors in KB tumor cell models [137]. Similarly, polymer-lipid hybrid nanoparticles conjugated with anti-EGFR antibodies were designed to enhance doxorubicin delivery to hepatocellular carcinoma, resulting in improved in vivo cytotoxicity and reducing the required drug dose by approximately sixfold compared with the nanoparticle-free formulation [138].

### Crossing biological barriers: blood–brain barrier and tumor penetration

Brain metastases and glioblastomas present significant challenges due to the restrictive nature of the blood–brain barrier (BBB). To address this, transferrin-functionalized AuNPs were developed for receptor-mediated transcytosis across the BBB. Using an acid-cleavable transferrin link, these nanoparticles achieved increased brain uptake compared with non-cleavable conjugates both in vitro and in vivo [139]. Two docetaxel-loaded PLGA nanoparticle formulations were developed using PRINT (Particle Replication in Nonwetting Templates) technology, a fabrication method for uniform cylindrical nanoparticles. PRINT-docetaxel and the acid-labile prodrug PRINT-C2-docetaxel were tested in an NSCLC murine model with brain metastases, achieving 13-fold and sevenfold higher intratumoral concentrations than small-molecule docetaxel, respectively. PRINT-C2-docetaxel further extended median survival by 35% compared to other treatments [140].

Pancreatic stroma represents an additional biological barrier to therapy. Using an arginine-glycine-aspartic acid (RGD) ligand to bind integrin αvβ3 expressed on tumor endothelium, researchers demonstrated that RGD-conjugated liposomes loaded with hydroxychloroquine and paclitaxel achieved greater stromal penetration and cytotoxicity than non-modified liposomes [141]. Another strategy explored the use of lymphocytes as potential drug carriers to overcome the bone marrow–blood barrier, a major challenge in bone tumor treatment. In an orthotopic bone metastasis model, aging neutrophils, which naturally home back to the bone marrow, were used to deliver cabazitaxel-loaded PLGA nanoparticles, achieving greater tumor growth inhibition compared with the free drug or neutrophil-free formulations [142].

### Multifunctional and theragnostic nanoparticles

Several studies have explored the integration of therapeutic and imaging agents into a single nanoplatform. Vascular endothelial growth factor-121-conjugated mesoporous silica nanoparticles designed for targeted positron emission tomography (PET) imaging and sunitinib delivery improved drug localization and imaging clarity in glioblastoma [143]. Gold nanoparticles conjugated with zinc phthalocyanine were tested in colorectal and breast cancer models for photodynamic therapy, demonstrating enhanced singlet oxygen generation and targeted phototoxicity [144]. Additionally, NIR-resonant silica-gold nanoshells (AuNSs) were compared with solid gold nanoparticles (AuNPs) for photothermal therapy and demonstrated superior heat generation and early treatment monitoring via PET imaging [145].

### Overcoming multidrug resistance

Multidrug resistance remains a major hurdle in chemotherapy, with drug-efflux pumps like P-glycoprotein playing a central role in limiting intracellular drug accumulation. One strategy to overcome this involves using NIR irradiation to cause ROS-mediated mitochondrial damage, thus disrupting the ATP production necessary for efflux activity. A PEGylated graphene oxide nanoplatform loaded with paclitaxel successfully reversed drug resistance in paclitaxel-resistant gastric cancer cells (HGC-27/PTX) by impairing oxidative phosphorylation, depleting ATP, and inhibiting P-glycoprotein function, leading to increased intracellular paclitaxel retention [146].

Due to their natural membrane composition, exosomes act as “Trojan horses” to defeat multidrug resistance. Exosome-encapsulated paclitaxel (exoPTX), derived from murine macrophages, increased cytotoxicity more than 50-fold in multidrug-resistant cancer models. When administered via the airway in a pulmonary metastasis mouse model, exoPTX achieved near-complete co-localization with lung lesions and significantly inhibited tumor progression compared with both paclitaxel alone and untreated controls [147].

Another approach employs PLGA and PLGA-PEG nanoparticles with decreased non-specific adhesivity, thus ensuring receptor-specific targeting while maintaining high diffusivity in the brain microenvironment [148].

## In Vitro Nanomedicine-based Gene Modulation

Incorporating gene therapy into nanomedicine has shown promise in regulating tumor progression. Amine-functionalized hydroxyapatite nanoparticles conjugated with anti-angiogenesis plasmid were used for gene therapy in breast cancer models, demonstrating efficient plasmid condensation, high transfection efficiency, and reduced angiogenesis [149]. Some investigators developed a polyethyleneimine-functionalized graphene oxide hydrogel for in situ transforming RNA nanovaccine delivery, leading to improved antigen presentation, enhanced CD8 + T-cell activation, and long-term immunity against cancer [150].

Another gene therapy approach leverages RNA interference to modulate gene expression. siRNAs inhibit gene transcription [151], while miRNAs regulate mRNA translation [152]. Doxorubicin-loaded AuNPs (Dox-Bcl2-AuNPs) conjugated with siRNAs targeted the anti-apoptotic gene Bcl-2, significantly reducing its expression in triple-negative breast cancer cells and enhancing clonogenic survival [153]. Similarly, MiR-124a, a pro-apoptotic FOXA2 down-regulator, encapsulated in mesenchymal stem cell-derived exosomes, significantly reduced glioblastoma cell viability in vitro and prolonged the median overall survival in paclitaxel models [154].

### pH-responsive and stimuli-sensitive drug release

Nanoparticles engineered for controlled drug release have been widely explored. A bio-metal–organic framework coated with chitosan was designed for pH-responsive doxorubicin release in breast cancer, demonstrating a slow, continuous release at physiological pH (7.4) but a significantly higher release (93%) in the TME (pH 6.8) [155]. Similarly, iron oxide nanoparticles coated with β-cyclodextrin and PEG were employed for 5-fluorouracil delivery, ensuring higher drug release at pH 6.8 while sparing normal cells [156]. Another triggered release strategy involves thermoacoustic therapy combined with single-walled carbon nanotubes for deep-seated tumors. In an orthotopic liver tumor model, nanotube injection followed by ultrashort microwave pulses generated thermoacoustic shockwaves, leading to mitochondrial damage, apoptosis, tumor growth inhibition, and extended survival [157].

### Overcoming tumor resistance to immunotherapy

Some investigators have reported on the use of nanoparticles to overcome resistance to immunotherapy, which remains a major challenge, particularly in the treatment of liver metastases, where activated hepatic stellate cells suppress T-cell infiltration and promote tumor growth by activating M2 macrophages and myeloid-derived suppressor cells. Relaxin (RLN), an antifibrotic peptide, deactivates activated hepatic stellate cells, reversing fibrosis and restoring immune function. In murine models of colorectal cancer, RLN-loaded lipid-calcium phosphate nanoparticles (RLN-LCPs) improved immune infiltration into liver metastases and prolonged survival both alone and in combination with PD-L1 blockade. Notably, gender differences were observed, with females showing a better response, likely due to 4.7-fold higher levels of endogenous RLN [158]. Another strategy aimed to reprogram hepatic sinusoidal endothelial cells to support anti-tumor immunity by leveraging α-melittin-conjugated NPs. α-Melittin, a peptide derived from bee venom, has been shown to induce the release of pro-inflammatory cytokines from endothelial cells. Compared with placebo, α-melittin-NPs significantly reduced the metastatic burden in the liver and prolonged survival across multiple in vivo models, including melanoma, TNBC, and colorectal cancer [159].

The tumor microbiome acts as a potent immunomodulator, driving immune suppression through molecules like lipopolysaccharides. In a murine colorectal cancer model, lipopolysaccharide-binding protein-loaded nanoparticles significantly increased CD8 + and CD4 + T-cell infiltration, reduced myeloid-derived suppressor cells, and improved survival. Outcomes were further enhanced when the nanoparticles were combined with immune checkpoint inhibitors. New vaccine strategies are also under investigation. The intranasally delivered CAP-Flu platform, an attenuated influenza A virus conjugated with the CpG immune adjuvant, improved dendritic cell activation and reduced lung metastases in in vivo melanoma models.

## Diagnostic applications of nanotechnology

Nanotechnology has revolutionized the field of oncology by enhancing imaging and diagnostic capabilities. Nanoparticles, due to their unique optical, magnetic, and electronic properties, serve as excellent contrast agents in various imaging modalities, such as magnetic resonance imaging (MRI), computed tomography (CT), PET, and optical imaging. For instance, SPIONs are widely used in MRI to improve the contrast of tumors, allowing for more precise disease localization and characterization [160]. AuNPs have also been extensively studied for their ability to enhance contrast in CT scans and provide high-resolution images due to their high atomic number and electron density, which increase photon absorption. Unlike conventional iodine-based agents, AuNPs offer prolonged circulation times and can be functionalized for targeted imaging [161].

In addition to improving imaging quality, nanotechnology facilitates the development of multifunctional theragnostic platforms that combine imaging and therapeutic features. For instance, quantum dots are semiconductor nanocrystals that emit fluorescence upon excitation, making them highly effective for in vitro and in vivo bioimaging​. Their engineered shell structure allows for easy surface functionalization, facilitating the conjugation of targeting ligands and therapeutic agents, thus enabling real-time monitoring of drug delivery and treatment response [162, 163].

Furthermore, the integration of nanotechnology in liquid biopsy has improved non-invasive cancer diagnostics, allowing for the detection of tumor biomarkers in body fluids such as blood and urine [164]. Circulating tumor DNA (ctDNA) and exosomes have emerged as promising cancer biomarkers, providing valuable genetic and molecular information on tumor progression, drug resistance, and metastasis [165, 166]. Exosomes, small extracellular vesicles carrying tumor-derived proteins and RNA, provide a rich source of biomarkers that can be analyzed using advanced nanotechnology-based sensors, enhancing early cancer detection and therapeutic decision-making [164].

One of the biggest challenges is effectively distinguishing between cancerous and healthy tissues, which can be achieved by detecting cancer-associated genetic mutations. Refining nanotechnology-based methods is crucial to improving sensitivity and specificity, especially in tumors and disease settings with low ctDNA shedding [167–169]. Recent advances in nanoplasmonic biosensors and microfluidic platforms have significantly improved the sensitivity of ctDNA and exosome-based cancer diagnostics [165]. For instance, AuNPs exhibit surface plasmon resonance (LSPR) features, where free electrons on the metal surface oscillate collectively in response to incident light, modifying optical absorption [170]. When ctDNA binds to functionalized AuNPs, this interaction shifts the optical signal, enabling real-time detection without additional costly procedures. These properties are further enhanced by the geometry of the nanoparticles. Due to their sharp tips, bipyramid-shaped AuNPs have shown superior sensitivity compared with rod-shaped AuNPs, allowing the detection of even low concentrations of KRAS G12D ctDNA in serum [170]. By integrating novel nanoparticle-based detection strategies with these emerging liquid biopsy approaches, the future of cancer diagnostics will likely shift toward real-time, minimally invasive monitoring, significantly improving early intervention and treatment outcomes.

Despite the significant advancements in nanotechnology for oncology diagnostics, several crucial gaps must be addressed. One major challenge is ensuring the biocompatibility and long-term safety of nanoparticles, which requires comprehensive studies on their pharmacokinetics, biodistribution, and potential toxicity in humans [171].

### Challenges in nano-based drug delivery systems

Although nano-based drug delivery systems (NDDS) have great potential to transform oncology, their clinical adoption is complicated by challenges ranging from formulation and stability issues to regulatory and ethical concerns (summarized in Table 4). Addressing these issues is crucial to unlocking the full potential of nanotechnology in cancer treatment.

**Table 4:** Current challenges in nanoparticle-mediated drug delivery for cancer treatment

Category	Challenge	Description
Design and Development	Nanoparticle Stability [241, 242]	Ensuring stability of nanoparticles during storage and administration is crucial as instability can cause aggregation or premature drug release
	Drug Loading Efficiency [243, 244]	Achieving a high drug loading capacity while maintaining the structural integrity of the nanoparticles presents a significant challenge
	Scalability and Reproducibility [245, 246]	It is challenging to manufacture nanoparticles on a large scale with consistent quality and performance
	Targeting Specificity [247, 248]	Developing nanoparticles that can precisely target cancer cells without affecting healthy cells remains a significant challenge
	Controlled Release [249, 250]	Achieving controlled and sustained drug release at the target site is crucial yet challenging
Biological Interactions	Immune System Evasion [251, 252]	Nanoparticles need to avoid detection and clearance by the immune system in order to effectively reach the tumor
	Biodistribution and Accumulation [253]	Ensuring that nanoparticles accumulate in the tumor, rather than in non-target tissues, is a major challenge
	Biocompatibility and Toxicity [254]	Nanoparticles must be biocompatible and non-toxic to prevent adverse reactions in the body
	Heterogeneous Tumor Microenvironment [255, 256]	The variability in tumor microenvironments can impact the penetration and efficacy of nanoparticle-based treatments
Regulatory Issues	Standardization and Characterization [257]	It is important to develop standardized methods for characterizing nanoparticles to obtain regulatory approval, but this process can be quite complex
	Safety and Efficacy Testing [184, 258, 259]	To ensure greater clinical reproducibility, preclinical safety and tolerability studies must follow a more extended and rigorous evaluation timeline, which negatively impact on costs
	Regulatory Pathways [260, 261]	Understanding and navigating the regulatory pathways for the approval of nanoparticle-based therapies can be a complex and uncertain process
Clinical Translation	Translation from Preclinical to Clinical [184]	Human metabolism is more complex than that of animal models, involving the reticuloendothelial, immune, and lymphatic systems as additional clearance pathways beyond the renal and hepatic routes
	Patient Variability [262]	Variations in genetics, disease state, and treatment response can impact nanoparticle-based therapy efficacy
	Cost and Accessibility [263]	High development and production costs can restrict patient access to nanoparticle-based therapies
	Integration with Existing Therapies [264, 265]	Combining nanoparticle-based therapies with existing cancer treatments requires careful consideration to avoid interactions and optimize therapeutic outcomes

### Biocompatibility and toxicity concerns

A major challenge in NDDS is ensuring that nanomaterials are biocompatible and do not elicit unpredictable adverse events in biological systems. The small size and high surface area of nanoparticles can lead to unintended toxicity, immunogenicity, or unexpected biodistribution, necessitating thorough preclinical and clinical evaluations. To overcome this challenge, more rigorous testing protocols are required [172].

Inorganic and carbon-based NPs potentially disrupt organ function due to persistent retention [173]. For instance, carbon nanotubes have been shown to induce hepatotoxicity (e.g., hepatocyte swelling, necrosis) [174], asbestos-like pulmonary inflammation and granuloma formation [175], and cardiovascular toxicity, including endothelial injury, myocardial fibrosis, and atherogenesis [176]. To address this, stimuli-responsive, size-reducible NPs have been studied. Researchers developed AuNPs functionalized with single-stranded DNA and cytochrome C to enable pH-responsive aggregation in acidic tumor environments. This strategy improved the drug’s clearance by overcoming size-related glomerular filtration limitations while maintaining the large nanoparticle dimensions needed for optimal NIR absorption in the cancer lesions [177]. The biocompatibility of iron oxide nanoparticles can be significantly influenced by their morphology and surface properties, which can be optimized through controlled synthesis [178]. And the development of biodegradable nanomaterials can mitigate long-term toxicity concerns [42, 179, 180].

Advanced detection methods, such as machine learning models, genotoxicity testing, and organ-on-a-chip (i.e., three-dimensional platforms) technologies, can help monitor the behavior of nanoparticles in dynamic biological environments. These tools provide predictive insights into nanoparticle toxicity, enabling rapid optimization of designs for clinical translation [179, 181, 182]. For instance, to address the discrepancies between the animal and the more heterogeneous human EPR effect, a recent study used an image segmentation machine learning model (nano-ISML) to map the distribution of ferritin nanocages loaded with doxorubicin across 32 tumor types. By analyzing and integrating data from over 67,000 tumor blood vessels, the model identified precise permeability parameters, enabling the refinement of nanoparticle designs to enhance their delivery potential [183].

Finally, long-term in vivo NDDS studies are lacking. Prolonged NDDS pharmacokinetic monitoring would improve the prediction of variable biodistribution across organs, better reflecting human clearance mechanisms where elimination pathways extend beyond renal and hepatic routes to involve the reticuloendothelial, immune, and lymphatic systems [184].

### Drug loading and release kinetics

Achieving optimal drug loading efficiency and ensuring a predictable release profile are critical for NDDS success. The optimization of nanoparticle drug loading must consider physicochemical compatibility (e.g., SLNs may fail to encapsulate hydrophilic compounds due to their lipophilic core [185]) and the election of an appropriate loading protocol, as seen with exosomes, where passive incubation leads to poor uptake and electroporation can damage membranes and induce cargo aggregation [186, 187]. Uncontrolled or premature drug release can reduce therapeutic efficacy and increase off-target effects [188]. Furthermore, the functionalization of NPs with specific ligands improves tumor targeting by promoting receptor-mediated uptake. Building on this strategy, researchers have extensively explored the integration of stimuli-responsive mechanisms to further enhance intratumoral drug release. The advantages and disadvantages of these approaches are summarized in Table 5. Endogenous triggers, such as the acidic pH of the TME or elevated intracellular glutathione levels, can activate drug release, leveraging the NP's cleavable linkers or redox-sensitive conjugates [189]. For instance, FePt NPs (i.e., IONs) surface-modified with cysteine exploit the high hydrogen peroxide content of the TME to catalyze Fenton-like reactions, triggering ROS-mediated apoptosis. In a lung cancer mouse model, these NPs significantly enhanced the effects of cisplatin and radiotherapy, leading to tumor volume reduction without additional systemic toxicity [190]. In parallel, exogenous stimuli, including light (PTT/PDT), ultrasound, or magnetic fields, enable on-demand control of drug release at the tumor site [191](Table 3**)**. To overcome their individual limitations, multi-stimuli NDDS platforms have emerged, integrating both endogenous and exogenous trigger technology to achieve enhanced selectivity, spatiotemporal control, and real-time treatment monitoring. For example, a hyaluronic acid-coated Fe(III)-tannic acid nanoparticle (FeIIITA@HA) was designed for the treatment of squamous cell carcinoma. This system combines CD44-targeted delivery with enzymatic degradation by tumor-associated hyaluronidase, promoting site-specific release and triggering both ferroptosis and apoptosis. Furthermore, the Fe(III)-tannic acid complex exhibits strong photothermal conversion efficiency under near-infrared light irradiation, enabling MRI-guided PTT. In vivo, this nanoplatform effectively suppressed tumor growth and demonstrated favorable biosafety due to its gradual biodegradation and clearance [192].

**Table 5:** Characteristics of Stimuli-Responsive Nanoparticles

Stimulus	Mechanism	Advantages	Limitations	Example
pH and enzyme-sensitive (endogenous)	Acidic TME, MMPs, and intracellular GSH trigger cleavage of sensitive linkers, enabling controlled drug release	Enhances site-specific activation, drug retention, and deep tumor penetration; reduces systemic toxicity	Enzyme levels and acidic pH can also be present in non-cancerous inflamed or infected tissues, risking off-target activation	Dual-sensitive Dendrimer-Dextran nanoparticles with MMP/pH-cleavable linker in GBM [266]
Redox (endogenous)	Tumor H₂O₂ activates FePt-mediated Fenton reaction, generating ROS and inducing oxidative stress	Enhances chemo- and radiosensitization via ROS-induced apoptosis and DNA damage	Effectiveness may be reduced by tumor antioxidant defenses (e.g., catalases)	Cysteine-coated FePt NPs with cisplatin/radiotherapy in NSCLC [190]
Light -PTT/PDT (exogenous)	NIR or visible light triggers heat generation (PTT) or ROS production (PDT) by photothermal agents or photosensitizers	Enables precise, non-invasive, on-demand tumor ablation; can induce immune activation	Limited penetration depth (PTT); efficacy depends on oxygen presence (PDT); risk of collateral damage to surrounding tissues	PDT: AuNP–antibody conjugates for CSC-targeted lung cancer therapy [231] PTT: Acid-functionalized MWCNTs eradicate breast tumors and promote immune cell infiltration [235]
Magnetic field (exogenous)	Alternating magnetic field induces localized heating of SPIONs (magnetothermal effect), also triggering drug release	Enables deep, non-invasive control of release; potential for combined hyperthermia and ROS-mediated therapy	Requires external magnetic setup; heat dissipation must be tightly controlled	FA-functionalized Fe₃O₄ SPIONs with DOX achieve targeted chemo-hyperthermia in MCF-7 cells [267]
Ultrasound (exogenous)	High-intensity focused ultrasound (HIFU) induces thermal ablation and cavitation	Enables deep tissue penetration, non-invasive ablation, and enhanced drug delivery; permits the use of sonodynamic agents (e.g., hematoporphyrin monomethyl ether, HMME) which produce ROS upon ultrasound exposure	Limited by tissue barriers (bone/gas), energy dispersion, and potential off-target heating	PFP/HMME-loaded PLGA nanoparticles enhance HIFU ablation via cavitation and sonodynamic synergy in breast cancer models [268]

### Biological barriers and clearance mechanisms

The inability to cross biological barriers such as mucosal layers, the BBB, and the mononuclear phagocyte system can lead to the elimination of nanoparticles before they reach their targets. Surface modifications of nanoparticles in NDDS such as PEGylation and ligand-mediated targeting improve circulation time and specificity. PEGylation enhances solubility, reduces immunogenicity, and prolongs bloodstream retention [193, 194]. PEGylated liposomes, for instance, improve hydrophobic drug delivery and stability [195] while also mitigating hemolytic toxicity [196]. However, anti-PEG antibodies can accelerate nanoparticle clearance, reducing efficacy [197, 198]. Exploring alternative surface modifications and optimizing spatially decoupled PEGylation can enhance targeting while minimizing unwanted interactions [199, 200]. Ligand-mediated targeting further enhances specificity by binding to overexpressed receptors, improving drug accumulation at target sites [56, 201]. Research on the immunogenicity of PEG and the development of innovative targeting strategies will be crucial for the successful translation of these technologies into clinical practice.

### Stability and scalability issues

One of the major hurdles in NDDS development is ensuring the stability of nanoparticles during storage and transportation. Nanoparticles often tend to aggregate, leading to changes in their physicochemical properties, which can compromise their efficacy. Optimizing formulation parameters, such as particle size and surface charge, can significantly improve stability [202]. Techniques like lyophilization have been shown to enhance the stability of nanoparticles, allowing for better preservation of their therapeutic properties [203]. Appropriate packaging and storage conditions are vital to maintain the efficacy of these formulations over time. Cryoprotectants like trehalose have been investigated to enable the long-term storage of exosome-based NPs, which typically require storage at –80 °C to preserve their structural integrity [204]. Additionally, large-scale manufacturing with batch-to-batch consistency remains a challenge. Accuracy in particle size, surface charge, and drug encapsulation efficiency is crucial to ensure the quality, efficacy, and safety of the manufactured product. A fundamental challenge in achieving the desired characteristics of drug delivery systems is optimization of synthesis methods such as single-emulsion solvent evaporation and nanoprecipitation. The amount of encapsulated material, stabilizer (e.g., PVA), and polymer concentration, and the organic-to-aqueous phase ratio, affect the size and encapsulation efficiency of the NPs [205]. Optimal performance is achieved when particles are kept within the 100–300 nm range and possess a zeta potential above −15 mV, which improves both delivery and biological interaction [206]. To overcome the complexity and rigidity of conventional manufacturing processes for PEGylated liposomes such as Doxil and Caelyx, microfluidic-based production systems have been developed. Automated platforms streamline the production process, facilitating large-scale production of PEGylated liposomal nanoparticles with quality comparable to the FDA-approved formulations [207]. The use of these approaches may scale up production without compromising quality.

### Limited translation from bench to bedside: regulatory and ethical challenges

Despite the remarkable results of NDDS in preclinical settings, their clinical translation remains limited. Challenges such as variability in nanoparticle synthesis, scalability, and batch reproducibility pose significant obstacles, and the underdeveloped regulatory frameworks for nano-based therapeutics further delay approval [208]. Standardizing nanoparticle synthesis protocols and implementing robust quality control measures are essential for consistency and reproducibility. Clear guidelines and thorough risk assessments are needed to address regulatory challenges and environmental impacts. Improved collaboration among academia, industry, and regulatory agencies could accelerate the development of standardized guidelines. And ethical concerns about the potential misuse and environmental impact of nanotechnology must be addressed to foster public trust and acceptance.

### Economic and logistical challenges

High production costs and complex manufacturing processes hinder the widespread adoption of NDDS. Integrating nanotechnology into existing treatment protocols demands a significant investment in infrastructure and workforce training. Cost-effective synthesis techniques, such as self-assembly and green chemistry, could help mitigate these expenses. Additionally, strategic partnerships between pharmaceutical companies and healthcare providers would facilitate clinical implementation and attract more scientific and financial resources. Educating clinicians and researchers on nanomedicine’s benefits and limitations will further promote its acceptance in mainstream oncology.

## Conclusion

This review summarizes the technological advances of NPs, highlighting the translation of preclinical nanotechnology discoveries into clinical applications that include clinical trials in oncology. Nanomedicine has made significant strides in optimizing pharmacokinetics and reducing adverse effects, enabling targeted treatment with improved efficacy and safety profiles, and it has the potential to continue improving cancer therapy via novel targeted drug delivery. Despite these advancements, challenges remain, including overcoming drug resistance, addressing biological barriers, and navigating regulatory complexities. Overcoming these hurdles will require continued interdisciplinary research, advanced clinical trials, and strategic integration of emerging technologies, such as artificial intelligence, to enhance therapeutic precision and patient outcomes. Preclinical studies of nanotechnology-based drugs have shown significant promise in improving drug efficacy, targeted delivery, and safety. These nanocarriers enhance tumor specificity, cross biological barriers, and offer multifunctional capabilities, including imaging and therapy. With further optimization, these approaches could revolutionize cancer treatment and pave the way for clinical translation.

The biocompatibility of nanomaterials represents a critical bottleneck for their clinical translation. To avoid unpredictable adverse reactions and life-threatening organ dysfunction in human systems, more predictive preclinical pharmacokinetic models are imperative. The effort to create increasingly reliable preclinical models must align with recent advancements towards three-dimensional platforms, such as organ-on-a-chip and other microphysiological systems. The fusion of nanotechnology with personalized medicine promises a future where cancer treatment is not only more effective but also tailored to individual patients, thereby maximizing therapeutic impact while minimizing off-target effects. The potential for co-delivery systems, theranostic platforms, and biomarker-driven diagnostics reinforces the critical role of nanomedicine in advancing cancer therapy. As research progresses, nanoparticle-based innovations and patient-centered approaches are likely to shape a new frontier in oncology, offering renewed hope and improved quality of life for cancer patients worldwide.

## Future perspectives in nanomedicine

Nanomedicine continues to redefine cancer therapy by advancing nanoparticle design for precise targeting, personalized treatment, and reduced toxicity. Nanoparticle-based drug delivery systems hold the potential to revolutionize oncology by enabling highly targeted, minimally invasive, and more effective therapeutic strategies. Future directions in nanomedicine will focus on optimizing nanoparticle properties to overcome barriers such as drug resistance and biological obstacles like the BBB. Integrating artificial intelligence and machine learning with nanotechnology will expedite the development of precision oncology solutions, enabling personalized treatments tailored to individual patient profiles.

Personalized medicine, combined with nanotechnology, promises a tailored approach to cancer treatment, where therapies are adapted to each patient’s unique genetic and molecular profile. This shift maximizes treatment efficacy and minimizes off-target effects, allowing for a patient-centered approach that reassures patients and their families. Additionally, co-delivery systems capable of delivering multiple therapeutic agents within a single nanoparticle foster synergistic effects, which further improve treatment outcomes. Theranostic platforms, integrating therapeutic and diagnostic functions, allow for real-time monitoring and dynamic adjustment of treatments, shaping a new era in cancer care that enhances both treatment precision and patient outcomes.

Nanotechnology also plays a crucial role in identifying and utilizing cancer biomarkers for early detection, prognosis, and treatment response monitoring. Advanced nanoscale materials and devices enable the detection of biomarkers at ultra-low concentrations with remarkable specificity and sensitivity, facilitating early cancer diagnosis and the development of targeted therapies. The application of nanotechnology in biomarker discovery and validation holds significant promise for enhancing the precision and efficacy of cancer therapies. Through sustained innovation and interdisciplinary research, nanotechnology is poised to further refine cancer treatments, offering a more promising, patient-centered future in oncology.

## Data Availability

No datasets were generated or analysed during the current study.

## Abbreviations

3D: Three-Dimensional
3 T3: Fibroblast Cell Line
5-FU: 5-Fluorouracil
ABI-009: Nab-Sirolimus
AIDS: Acquired Immunodeficiency Syndrome
AML: Acute Myeloid Leukemia
ATC: Anaplastic Thyroid Cancer
ATP: Adenosine Triphosphate
Ab: Antibody
AlPcS₄Cl: Aluminum Phthalocyanine Chloride
AuCl₄: Tetrachloroaurate Ion
AuNPs: Gold Nanoparticles
BBB: Blood–Brain Barrier
BCG: Bacillus Calmette-Guérin
BCLC: Barcelona Clinic Liver Cancer
BIND-014: Prostate-Specific Membrane Antigen-Targeted Docetaxel Nanoparticles
BS: β-Sitosterol Caco-2
Bio-MOF: Bio-Metal–Organic Framework
CBDCA: Carboplatin
CDDP: Cisplatin
CI: Confidence Interval
CMC: Critical Micelle Concentration
CNT: Carbon Nanotube
CR: Complete Response
CRC: Colorectal Cancer
CRLX101: Camptothecin-Loaded Polymeric Nanoparticle
CRT: Chemoradiotherapy
CS: Chitosan
CT: Computed Tomography
Cu MOF: Copper Metal–Organic Framework
ctDNA: Circulating Tumor DNA
DCR: Disease Control Rate
DCR-MYC: MYC-Targeting siRNA in Lipid Nanoparticles
DFS: Disease-Free Survival
DLT: Dose-Limiting Toxicity
DOPC: Dioleoylphosphatidylcholine
DPPC: Dipalmitoyl Phosphatidylcholine
Den: Dendrimer Nanoparticles
DoR: Duration of Response
EFS: Event-Free Survival
EGFR: Epidermal Growth Factor Receptor
EMA: European Medicines Agency
EPR: Enhanced Permeability and Retention
FA: Folic Acid
FDA: Food and Drug Administration
FEC: Fluorouracil, Epirubicin, Cyclophosphamide
FNPs: Iron Oxide Nanoparticles
FRA: Folate Receptor-Alpha
FUS: Focused Ultrasound
FeNPs: Iron Oxide Nanoparticles
Fe₃O₄: Iron Oxide
Fe₃O₄ NPs: Magnetic Iron Oxide Nanoparticles
G ≥ 3 AE: Grade 3 or Higher Adverse Events
GBM: Glioblastoma
GSH: Glutathione
GO: Graphene Oxide
H1299: Lung Cancer Cell Lines
H_2_O_2_: Hydrogen Peroxide
HCC: Hepatocellular Carcinoma
HER2: Human Epidermal Growth Factor Receptor 2
HMME: Hematoporphyrin Monomethyl Ether
HNSCC: Head and Neck Squamous Cell Carcinoma
HR: Hazard Ratio
HSPC: Hydrogenated Soy Phosphatidylcholine
HepG2: Liver Cancer
HfO₂: Hafnium Oxide
HIFU: High-Intensity Focused Ultrasound
IC50: Inhibitory Concentration 50%
ICIs: Immune Checkpoint Inhibitors
IO: Immunotherapy
IONPs: Iron Oxide Nanoparticles
LAR: Long-Acting Release
LHRH: Luteinizing Hormone-Releasing Hormone
LNPs: Lipid Nanoparticles
LTLD: Lyso-Thermosensitive Liposomal Doxorubicin
LY01610: Liposomal Irinotecan Formulation
mFOLFOX: Modified FOLFOX Chemotherapy Regimen (Fluorouracil, Leucovorin, Oxaliplatin)
miRNA: MicroRNA
MMP: Matrix Metalloproteinaise
mOS: Median Overall Survival
mPFS: Median Progression-Free Survival
mRFS: Median Relapse-Free Survival
mRNA: Messenger Ribonucleic Acid
mTOR: Mammalian Target of Rapamycin
MPR: Major Pathological Response
MRI: Magnetic Resonance Imaging
MSNs: Mesoporous Silica Nanoparticles
MTD: Maximum Tolerated Dose
MWCNTs: Multi-Walled Carbon Nanotubes
MYC: Myelocytomatosis Viral Oncogene Homolog
MoAb: Monoclonal Antibody
NC-6004: Cisplatin-Containing Polymeric Nanoparticle
NIR: Near-Infrared
NK105: Micellar Paclitaxel Formulation
NKTR-102: PEGylated Irinotecan
NSCLC: Non-Small Cell Lung Cancer
Nab-Paclitaxel: Nanoparticle Albumin-Bound Paclitaxel
Nab-Sirolimus: Albumin-Bound Sirolimus
ORR: Overall Response Rate
OS: Overall Survival
PBS: PhosphateBuffered Saline
pCR: Pathological Complete Response
PDT: Photodynamic Therapy
PE: Primary Endpoint
PEG: Polyethylene Glycol
PET: Positron Emission Tomography
PFS: Progression-Free Survival
PLGA: Poly(Lactic-co-Glycolic Acid)
PO: Primary Outcome
PR: Partial Response
PSMA: Prostate-Specific Membrane Antigen
PXT: Paclitaxel
PTT: Photothermal Therapy
RES: Reticulo-Endothelial System
RFS: Relapse-Free Survival
RNA: Ribonucleic Acid
RNAi: RNA Interference
ROS: Reactive Oxygen Species
RP2D: Recommended Phase 2 Dose
RT: Radiotherapy
SAE: Serious Adverse Event
SCLC: Small Cell Lung Cancer
SD: Stable Disease
siRNA: Small Interfering RNA
SPARC: Secreted Protein, Acidic and Rich in Cysteine
SPIONs: Superparamagnetic Iron Oxide Nanoparticles
SWCNT: Single-Walled Carbon Nanotube
TME: Tumor Microenvironment
US: Ultrasound
